# Metabolites of Prickly Rose: Chemodiversity and Digestive-Enzyme-Inhibiting Potential of *Rosa acicularis* and the Main Ellagitannin Rugosin D

**DOI:** 10.3390/plants10112525

**Published:** 2021-11-20

**Authors:** Daniil N. Olennikov, Vladimir V. Chemposov, Nadezhda K. Chirikova

**Affiliations:** 1 Laboratory of Medical and Biological Research, Institute of General and Experimental Biology, Siberian Division, Russian Academy of Science, 670047 Ulan-Ude, Russia; 2 Department of Biology, Institute of Natural Sciences, North-Eastern Federal University, 677027 Yakutsk, Russia; soulkage94@gmail.com (V.V.C.); hofnung@mail.ru (N.K.C.)

**Keywords:** *Rosa acicularis*, liquid chromatography–mass spectrometry, metabolomics, ellagitannins, flavonoids, rugosin D, simulated gastrointestinal digestion, α-amylase inhibitors

## Abstract

Prickly rose (*Rosa* *acicularis* Lindl.) is the most distributed rose species in the Northern Hemisphere, used by indigenous people for various food purposes. The lack of detailed information about the chemical composition of *R. acicularis* has led us to study the phytochemical composition and metabolic profile of prickly rose extracts using chromatographic techniques. Many groups of phenolic and non-phenolic compounds were quantified in the leaves, flowers, roots and fruits of *R. acicularis*. Phenolic compounds were the dominant phytochemicals in the aerial parts and roots of *R. acicularis*. A precise study by high-performance liquid chromatography with photodiode array detection and electrospray ionization triple quadrupole mass spectrometric detection showed the presence of 123 compounds, among which ellagic acid derivatives, ellagitannins, gallotannins, catechins, catechin oligomers, hydroxycinnamates and flavonoid glycosides of kaempferol, quercetin and dihydroquercetin were all identified for the first time. The most abundant phenolic compounds were ellagitannins and flavonoid glycosides, with a maximal content of 70.04 mg/g in leaves and 66.72 mg/g in flowers, respectively, indicating the great ability of *R. acicularis* organs to accumulate phenolic compounds. By applying a standardized static, simulated gastrointestinal digestion method, we found the inhibitory potential of the leaf extract against digestive α-amylases. A pancreatic α-amylase activity-inhibiting assay coupled with HPLC microfractionation demonstrated high inhibition of enzyme activity by ellagitannin rugosin D, which was later confirmed by a microplate reaction with mammalian α-amylases and the simulated digestion method. This study clearly demonstrates that *R. acicularis* leaf extract and its main component, ellagitannin rugosin D, strongly inhibit digestive α-amylase, and may be a prospective antidiabetic agent.

## 1. Introduction

*Rosa* is one of the largest genera of the Rosaceae family, and is an amazing plant genus that has found practical application by humans since ancient times. In a botanical sense, the genus includes about 400 species in the form of shrubs and semi-shrubs, widespread mainly in the Northern Hemisphere [1]. Disregarding the decorative value of the rose species, it should be noted that they are of great importance as medicinal and food plants. Well-known rose species include *R. canina*, *R. damascena*, *R. majalis* and *R. rugosa*, amongst others, which are sources of bioactive ellagitannins, flavonoids, triterpenoids, carotenoids and fatty oils that have antioxidant, antitumor, anti-inflammatory, gastroprotective and antiatherogenic activity [2]. It is not difficult to notice that the largest amount of scientific information on the chemical composition and biological activity of the rose species was created for the southern plants. Northern species growing in the territory of Siberia and the Far East are used less, due to the lack of scientific data and poor awareness about their positive properties. The promising northern species of wild rose include *R. acicularis*, *R. amblyotis*, *R. davurica* and *R. oxyacantha,* which inhabit large natural areas and are distinguished by high productivity [3].

**Figure 1:**
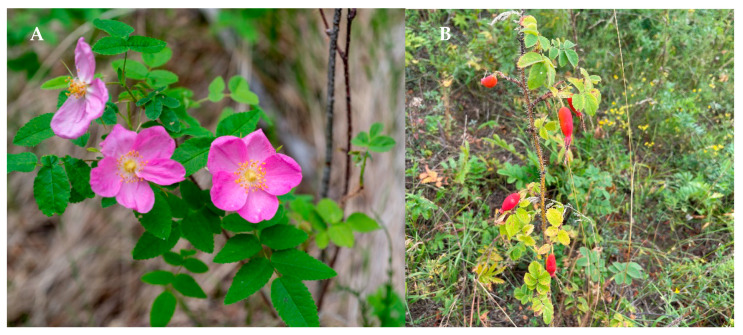
Prickly rose (*Rosa* *acicularis*) in its natural habitat, in the flowering stage (**A**) (Republic Buryatia, Selenginskii District) and the fruiting stage (**B**) (Republic Buryatia, Mukhorshibirskii District).

The largest territories of Siberia are occupied by *Rosa* *acicularis* Lindl., or prickly rose, which is a short shrub (up to 2 m) with brown branches that are densely covered with horizontally spaced spines and bristles [4]. Their bright green leaves consist of oblong–ovoid leaves seeded with glands, and the dark-pink single flowers have a characteristic pleasant smell (Figure 1). Elongated oval dark-red fruits ripen by the middle to the end of August and are harvested by the native population of the Siberian regions. This rose species is grown in mixed, sparse dark coniferous pine and birch forests, in meadows, along river banks, in woodlands and forest–tundra in all regions of Western, Central and Eastern Siberia, as well as the Far East, Central Asia, Mongolia, North China and North America [5]. Along with other rose species, such as *R. majalis* and *R. davurica*, the prickly rose forms the densely forested areas in Siberia, with a high fruit productivity (2.5–6 tonns/ha), and industrial harvesting is carried out for its fruits [6].

A wide distribution of *R. acicularis* has contributed to its use as a food and medicinal plant, and all the organs (leaves, flowers, roots and fruits) of the plant are of practical importance. The most common way to use *R. acicularis* is to brew it as a tea, the taste of which varies depending on the part of the plant used, from sweet and sour to tart and herbal [7]. Jams, syrups and compotes are prepared from the prickly rose fruits, characterized by good gelling properties. In the medical systems of Siberian and Asian peoples, rosehip medicines are used to treat diseases of the stomach and intestines, as an appetizing and anti-inflammatory agent, as well as in remedies to restore health after long-term illnesses [8]. Yakut traditional nomad medicine recommends the ripe fruits of *R. acicularis* to strengthen the gums, and an unripe fruit decoction is used to treat cardiac problems [9]. The decoction of the twigs of fresh bushes is a prophylactic remedy against diarrhea and intestinal diseases, and the tea of the leaves is used as a diuretic [10]. The Buryat lamas use the fruits of *R. acicularis* to treat diseases of *bile* and to suppress *wind* [11], as well as to destroy poisons and contribute to the growth of teeth [12]. The stem bark is applied as an antidote and used to cure lymphatic system diseases [13].

In official medical practice, rosehip is applied as a source of ascorbic-acid-containing concentrates and syrups and carotene-rich oils and creams. The most commonly used roses for commercial purposes are *R. rugosa*, *R. canina* and *R. majalis,* as evidenced by the good level of knowledge regarding them [2,14,15]. Information about the chemical composition and bioactivity of *R. acicularis* is limited.

The early study of *R. acicularis* leaves showed the good Fe-reducing power and antioxidant potential of the extract in radical scavenging assays against free radicals such as DPPH, ABTS and superoxide anion, caused by the presence of phenolic compounds (126 mg/g), flavonoids (8 mg/g) and flavanols (1 mg/g) in the plant [16]. Various *R. acicularis* extracts were effective as inhibitors of lipase activity [17] and HIV-1 protease activity [18], as well as antimicrobial agents [19]. The known scientific information about *R. acicularis* chemistry includes data on the organ-specific distribution of nine microelements [20], the essential oil and fatty acid composition [21] in addition to the total level of flavonoids, phenols and procyanidins in leaf isolates extracted by different solvents [22]. So far, however, there has been no precise study of *R. acicularis* metabolite composition, nor any LC-MS-based investigations of prickly rose extracts. Our earlier study of Siberian plants demonstrated the high potential of *R. acicularis* extracts to inhibit α-glycosidase, indicating the promising antidiabetic potential of prickly rose extracts [23], especially given the ethnopharmacological data about the use of *R. acicularis* decoction and tincture to treat diabetes in traditional medicine of Siberian nomads [13].

As part of an ongoing study on plant antidiabetic metabolites [23,24,25,26,27,28,29], and based on the preliminary information available concerning rose metabolites, we performed qualitative and quantitative chromatographic analyses of phenolic compounds for the first time in the leaves, flowers, roots and fruits of *R. acicularis* by means of high-performance liquid chromatography with photodiode array detection and electrospray ionization triple quadrupole mass spectrometric detection (HPLC-PDA-ESI-tQ-MS/MS). The total extracts of *R. acicularis* organs were bioassayed by in vitro methods for their ability to inhibit digestive enzymes, followed by an HPLC-based bioassay, which allowed metabolites with the greatest inhibitory potential to be found. Finally, rugosin D was found to be the main inhibitor of α-amylase in a simulated gastrointestinal digestion model.

## 2. Results and Discussion

### 2.1. Metabolites of Rosa acicularis: Distribution of Phytochemicals in Organs

The known ethnopharmacological data refer to the use of the whole plant of *R. acicularis* for medicinal and dietary purposes [17,18,19,20,21,22]. A preliminary study of the general phytochemical composition of *R. acicularis* showed the varying content of phenolic and non-phenolic compounds in different organs (Table 1). The leaves accumulated ellagitannins (73.69 mg/g of dry weight), gallotannins (21.23 mg/g) and hydroxycinnamates (1.47 mg/g), while high levels of flavonoids (67.39 mg/g as flavonols and 0.73 mg/g as dihydroflavonols), anthocyanins (5.34 mg/g) and water-soluble polysaccharides (65.14 mg/g) were found in flowers, and high levels of catechins (43.04 mg/g) and proanthocyanidins (26.04 mg/g) were found in root samples. The fruits were able to store free organic acids (42.59 mg/g; measured as titratable acids), ascorbic acid (56.12 mg/g), carotenoids (2.33 mg/g) and lipids (65.12). The total phenolic content of *R. acicularis* organs varied from 3.03 mg/g in fruits and 85.18 mg/g in roots to 160.75 mg/g in flowers and 173.98 mg/g in leaves. All this points to the organ-specific accumulation of phytochemicals in the whole *R. acicularis* plant.

Early data of *R. acicularis* phytochemicals showed a lower level of total phenolics (74 mg/g), flavonoids (24 mg/g) and proanthocyanidins (13 mg/g) in leaf extracts of Chinese origin [22]. The total phenolic content and flavonoid content of leaf extracts of the Turkish species *R. sempervirens* were 17–203 mg/g and 10–96 mg/g, respectively [30]. Analysis of leaves of 17 Polish *Rosa* species revealed variations in total phenolic content, from 5.7% (*R. rugosa*) to 15.2% (*R. canina* var. *dumalis*), and flavonoids, from 5.6 mg/g (*R. vosagiaca*) to 19.01 mg/g (*R. gallica*) [31]. Four Hungarian rosehips (*R. canina*, *R. gallica*, *R. rugosa* and *R. spinosissima*) contained 255.9 to 766.0 mg/100 g of total phenolics [32], and the fruits of four Lithuanian roses (*R. rugosa*, *R. pimpinellifolia*, *R. multiflora* and *R. canina*) showed a variation of 15–50 mg/g of total phenolics and 0.5–5 mg/g of flavonoids [33].

The study of the distribution of water-soluble sugars in *R. rugosa* organs found 0.2% in achenes, 0.4% in leaves, 0.8% in petals and 15% in fruits [34]. The ascorbic acid content was 274–1157 mg/100 g in Iranian rosehips [35] and 121–360 mg/100 g in Transylvanian *R. canina* fruits [36]. The level of carotenoids in fruits of Swedish species, such as *R. dumalis*, *R. rubiginosa* and *R. spinosissima,* was 0.3–1 mg/g [37]. In comparing the phytochemical composition of prickly rose with other *Rosa* species, we can deduce the remarkable level of valuable phenolics and non-phenolic compounds in *R. acicularis*.

### 2.2. Metabolites of Rosa acicularis: LC-MS Profile and Organ-Specific Distribution

The study of metabolite diversity in *R. acicularis* was realized using high-performance liquid chromatography with photodiode array detection and electrospray ionization triple quadrupole mass spectrometric detection (HPLC-PDA-ESI-tQ-MS/MS) in four plant organs: leaves, flowers, roots and fruits.

#### 2.2.1. Leaves

**Figure 2:**
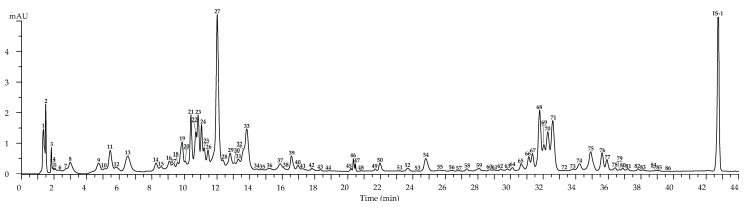
High-performance liquid chromatography with photodiode array detection (HPLC-PDA) chromatogram (270 nm) of *R. acicularis* leaf extract (July sample). Compounds are numbered as listed in Table 2. IS-1—3′,4′-di-*O*-acetyl-*cis*-khellactone (25 μg/mL).

Chromatograms of *R. acicularis* leaf samples collected in three developmental stages, May (the beginning of vegetation), July (blossom stage) and September (senile stage), showed the maximal diversity (86 compounds) in the July samples (Figure 2, Table 2). Ellagic acid and hexosides, ellagitannins, gallotannins, catechins, catechin oligomers, hydroxycinnamates and flavonoids (*incl*. quercetin glycosides, kaempferol glycosides and dihydroquercetin glycosides) were detected after comparing UV, mass spectral patterns and chromatographic behavior with reference standards and literature data [24,38,39,40,41,42,43,44,45,46,47,48,49].

Free ellagic acid (**29**), a common component of roses [2], was found in all samples of *R. acicularis* leaves, with the content of it rising from May (0.25 mg/g) to September (4.29 mg/g). Two non-methylated hexosides, 1-*O*-ellagoyl-β-D-glucopyranoside (**28**) and ellagic acid tri-*O*-hexoside (**6**), and six derivatives of ellagic acid methyl esters—monohexosides of ellagic acid *O*-methyl ester (**30**, **31**), ellagic acid di-*O*-methyl ester (**36**), di-ellagoyl *O*-methyl ester (**39**), di-ellagoyl di-*O*-methyl ester (**43**) and ellagic acid tri-*O*-methyl ester (**44**)—were identified as trace compounds. Some glycosides of ellagic acid were mentioned previously for *R. rugosa* and *R. canina* [2]. The total content of ellagic acid and its hexosides in *R. acicularis* leaves was lowest in the May samples (0.51 mg/g), rising in July (4.74 mg/g) and then showing a slight reduction in September (4.43 mg/g).

Leaf extracts of *R. acicularis* contained 12 ellagitannins, and the identity of 10 compounds were confirmed as tellimagrandin I_1_ (**14**), I_2_ (**19**), II (**21**; isomeric **23**), rugosin A (**26**), B_1_ (**16**), B_2_ (**22**), D (**27**), E_1_ (**24**) and E_2_ (**25**) using reference standards. Isomeric compounds **4** and **5** were described as ellagitannins due to their mass spectral patterns, with *m*/*z* 795, 633, 481, 463 and 301 typical for the galloyl-hexahydroxydiphenoyl-di-*O*-hexosides [41,42]. Ellagitannins with hexahydroxydiphenoyl and valoneoyl substituents are known tannins of the Rosaceae family [50] and some *Rosa* species (*R. canina* [51], *R. chinensis* [52] and *R. rugosa* [53]). The dominant ellagitannins of *R. acicularis* leaves were rugosin D, tellimagrandin II_1_ and tellimagrandin II_2_, with the highest levels of 41.15 mg/g, 8.98 mg/g and 8.29 mg/g, respectively, in summer samples. An increase in ellagitannin accumulation was found in *R. acicularis* leaves from spring to summer, followed by a decrease in the autumn. The latter involves the possibility of a seasonal destruction of polymeric ellagitannins by a specific tannase, resulting in the release of the simpler compound [54]. Ellagic acid, as a final product of ellagitannin cleavage, showed a marked increase in autumn samples of *R. acicularis* leaves, which supports this hypothesis. The total content of ellagitannins in *R. acicularis* leaves varied from 26.99 mg/g in May to 70.04 mg/g in July, and was much more than those in the leaves of *R. canina* (1.11 mg/g), *R. glauca* (1.05 mg/g), *R. sempervirens* (1.09 mg/g) and *R. rubiginosa* (4.81 mg/g) [55].

Gallic acid (**2**) was found in the leaves of *R. acicularis* in the free state and in the glycosidic form with different galloyl contents: one—1-*O*-galloyl-glucopyranoside or glucogallin (**1**); two—1,6-di-*O*-galloyl glucose (**13**) and isomer **12**; three—1,3,6-tri-*O*-galloyl glucose (**18**) and isomers **17** and **20**; four—1,2,3,6-tetra-*O*-galloyl glucose (**33**) and isomer **32**; five—1,2,3,4,6-penta-*O*-galloyl glucose (**47**) and isomer **46**; and six—hexa-*O*-galloyl hexoses **48**, **49** and **50**. Galloylated glycosides are still rarely described in rose plants containing mono-, di- and three-galloyl hexoses, such as *R. rugosa* and *R. canina* [2,55]. The main gallotannin of *R. acicularis* leaves was 1,2,3,6-tetra-*O*-galloyl glucose, with the highest level (10.54 mg/g) found in July samples. The accumulation pattern of all gallotannins in *R. acicularis* leaves was close to that of the ellagitannins: the increase period (spring–summer) bringing a sharp drop in autumn, owing to the destruction of polygalloylated glycosides to free gallic acid, which showed the rapid increase in content from 3.68 mg/g in July to 10.39 mg/g in September.

Flavonoids were the largest phenolic group of *R. acicularis* leaves, supplied by derivatives of kaempferol, quercetin and dihydroquercetin, all in the form of glycosides, and gave aglycone ions in the MS^2^ spectra with *m*/*z* 285, 301 and 303, respectively. The mass spectrometric analysis demonstrated the loss of carbohydrate fragments of glucose/hexose (162 a.m.u.), glucuronic acid/hexuronic acid (176 a.m.u.) and arabinose/pentose (132 a.m.u.), as well as acyl fragments of *p*-coumaric acid (146 a.m.u.) and gallic acid (152 a.m.u.).

The group of kaempferol glycosides included known compounds, such as kaempferol-3-*O*-glucuronoide (**71**), kaempferol-3-*O*-arabinoside (juglanin; **74**), kaempferol-3-*O*-(6″-*O*-*p*-coumaroyl)-glucoside (tiliroside; **75**) and kaempferol-3-*O*-(6″-*O*-galloyl)-glucoside (**76**), which were identified by comparison with reference standards as well as with 12 compounds with tentative structures. Flavonoids **71**, **74** and **75** were described in *R. canina* and *R. rugosa* [2].

The glycoside moiety of the non-acylated kaempferol glycosides were hexuronic acid and hexose, in ratios of 1:3 (**58**), 2:2 (**60**), 1:2 (**62**) and 1:1 (**69**), and hexuronic acid and pentose in a ratio of 1:1 (**70**). There are some known analogues of kaempferol glycosides with a hexuronic acid:hexose ratio of 1:1, such as kaempferol-3-*O*-glucoside-7-*O*-glucuronide from *Tulipa gesneriana* (Liliaceae) [56] and *Allium microdicityon* [57] in addition to kaempferol-3-*O*-glucuronide-7-*O*-glucoside from *Euphorbia sanctae-catharinae* (Euphorbiaceae) [58], as well as kaempferol glycosides with a hexuronic acid:hexose ratio of 1:2, such as kaempferol 3-*O*-sophoroside-7-*O*-glucuronide from *Allium cepa* (Alliaceae) [59] and kaempferol 3-*O*-gentiobioside-7-*O*-glucuronide from *Tulipa gesneriana* [56]. Compounds **58** and **60** have no possible structure candidates among the known flavonoids.

**Figure 3:**
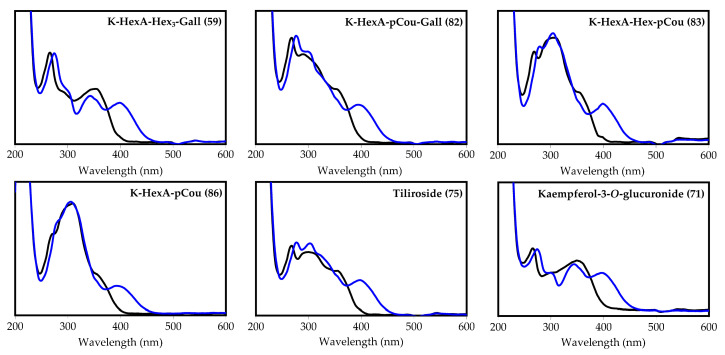
Ultraviolet absorption spectra of compounds **59** (kaempferol 3-*O*-hexuronoside-tri-*O*- hexoside-*O*-gallate), **82** (kaempferol 3-*O*-hexuronoside-*O*-*p*-coumarate-*O*-gallate), **83** (kaempferol 3-*O*-hexuronoside-*O*-hexoside-di-*O*-*p*-coumarate), **86** (kaempferol 3-*O*-hexuronoside-tri- *O*-*p*-coumarate), **75** (tiliroside), and **71** (kaempferol-3-*O*-glucuronide) before (black line) and after the addition of AlCl_3_ (blue line).

Acylated kaempferol glycosides showed specific UV absorbance, allowing us to determine the presence of acylation units linked with the flavonoid molecule (Figure 3). Moreover, all spectra of AlCl_3_ complexes included longwave maxima at 400 ± 4 nm, typical for 3-*O*-substituted kaempferol glycosides with free 5-*O*-, 7-*O*- and 4′-*O*-positions [60].

**Figure 4:**
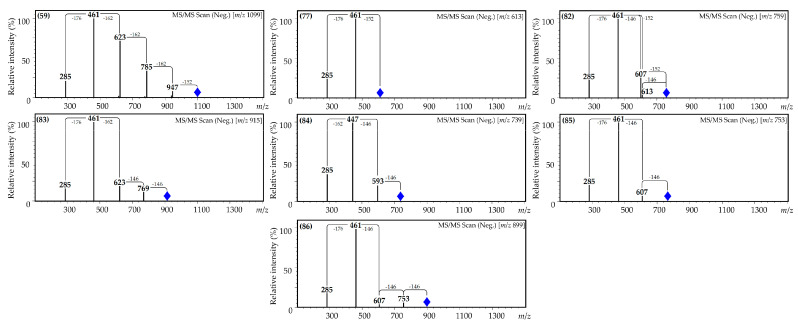
Mass spectra (MS^2^) of acylated kaempferol glycosides **59**, **77** and **82**–**86**.

Mass spectral behavior indicated various combinations of fragments in the non-aglycone part of the molecule, such as hexuronic acid:hexose:galloyl in a ratio of 1:3:1 (**59**), hexuronic acid:galloyl in a ratio of 1:1 (**77**), hexuronic acid:*p*-coumaroyl:galloyl in a ratio of 1:1:1 (**82**), hexuronic acid:hexose:*p*-coumaroyl in a ratio of 1:1:2 (**83**), hexose:*p*-coumaroyl in a ratio of 1:2 (**84**) and hexuronic acid:*p*-coumaroyl in ratios of 1:2 (**85**) and 1:3 (**86**) (Figure 4).

Compounds **59** and **77** demonstrated the hypsochromic shift of the kaempferol glycoside shoulder, from 287 nm to 284 nm, and the hyperchromic shift of the shortwave band, signaling the galloyl moiety attachment [46]. In mass spectra, we found the primary loss of the particle with *m*/*z* 152 related to the galloyl particle (*m*/*z* 1099 [M-H]^−^→947 for **59;** 613 [M-H]^−^→461 for **77**), followed by the loss of hexose (*m*/*z* 947 [M-H-galloyl]^−^→785, 623, 461 for **59**) and finally hexuronic acid (*m*/*z* 461→285 for **59** and **77**). The provisional structures of **59** and **77** were found to be kaempferol 3-*O*-(*O*-galloyl-tri-*O*-hexosyl)-hexuronoside and kaempferol 3-*O*-(*O*-galloyl)-hexuronoside, respectively. Both compounds have no analogues among the known kaempferol glycosides.

The UV pattern of compound **82** was close to the known coumaroylated flavonoid kaempferol-3-*O*-(6’´-*O*-*p*-coumaroyl)-glucoside (tiliroside; **75**), with the hyperchromed band II indicating the presence of an additional galloyl substituent [60]. The mass spectra of **82** showed the loss of galloyl (*m*/*z* 759 [M-H]^−^→607) and coumaroyl (*m*/*z* 759 [M-H]^−^→613) fragments, and was determined to be the unknown kaempferol 3-*O*-(*O*-*p*-coumaroyl-*O*-galloyl)-hexuronoside.

The absorption spectra of compounds **83**, **84** and **85** saved the kaempferol-3-*O*-glycoside spectral features, including band II (267 nm) and I (as a shoulder at 354 nm), but the intensity of the band at 310 nm—caused by the presence of the coumaroyl fragment—was much greater than mono-coumaroylated tiliroside. In the mass spectra, we found the loss of two fragments with *m*/*z* 146 (coumaroyl) for all three compounds: *m*/*z* 915 [M-H]→469, 623 for **83**; 739 [M-H]^−^→593, 447 for **84**; and 753 [M-H]^−^→607, 461 for **85**. Taking into account the spectral data, the possible structures of **83**, **84** and **85** were kaempferol 3-*O*-(di-*O*-*p*-coumaroyl-*O*-hexosyl)-hexuronoside, kaempferol 3-*O*-(di-*O*-*p*-coumaroyl)-hexoside and kaempferol 3-*O*-(di-*O*-*p*-coumaroyl)-hexuronoside, respectively. Only flavonoid **84** has possible analogues in characterized structures such as kaempferol 3-(2″,6″-di-*O*-*p*-coumaroyl)-glucoside from *Quercus canariensis* (Fagaceae) [47] or kaempferol 3-(3″,6″-di-*O*-*p*-coumaroyl)-glucoside from *Aerva lanata* (Amaranthaceae) [61].

Compound **86** has a UV pattern with a single maximum at 311 nm and two shoulders at 270 nm and 352 nm that look similar to the absorption spectra of mono- and di-coumaroylated kaempferol glycosides. The gradual loss of three fragments with *m*/*z* 146 was detected (*m*/*z* 899 [M-H]^−^→753, 607 and 461), which suggested that the flavonoid was tri-coumaroylated kaempferol glycoside or kaempferol 3-*O*-(tri-*O*-*p*-coumaroyl)-hexuronoside, but this is still unknown.

Fifteen quercetin derivatives were detected in *R. acicularis* leaves, such as the known compounds quercetin-3-*O*-rutinoside (rutin; **65**), quercetin-3-*O*-glucuronide (miquelianin; **68**), quercetin-3-*O*-rhamnoside (quercitrin; **72**) and quercetin-3-*O*-(6″-*O*-*p*-coumaroyl)-glucoside (helichrysoside; **73**). The remaining quercetins were non-acylated and acylated glycosides. In the glycosidic moiety of non-acylated glycosides, hexuronic acid and hexose were found in ratios of 2:3 (**52**), 1:3 (**54**), 2:2 (**57**), 1:2 (**61**) and 1:1 (**66**), as well as hexuronic acid and pentose in a ratio of 1:1 (**67**).

**Figure 5:**
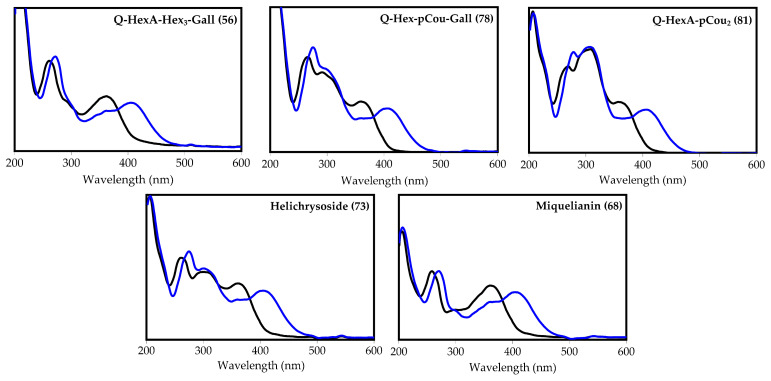
Ultraviolet absorption spectra of compounds **56** (quercetin 3-*O*-hexuronoside-tri-*O*-hexoside-*O*-gallate), **78** (quercetin 3-*O*-hexuronoside-*O*-*p*-coumarate-*O*-gallate), **81** (quercetin *O*-hexuronoside-di-*O*-*p*-coumarate), **73** (helichrysoside) and **68** (miquelianin) before (black line) and after the addition of AlCl_3_ (blue line).

The acylated quercetin derivatives included hexuronic acid, hexose and gallic acid (ratio of 1:3:1; **56**); hexose, gallic acid and *p*-coumaric acid (ratio of 1:1:1; **78**); hexuronic acid, gallic acid and *p*-coumaric acid (ratio of 1:1:1; **79**); hexose and *p*-coumaric acid (ratio of 1:2; **80**); and hexuronic acid and *p*-coumaric acid (ratio 1:2; **81**) in the glycosidic fragments. As in the case of acylated kaempferol glycosides, the UV patterns of quercetin glycosides varied with the type of acylation group, and the AlCl_3_ spectra were characterized by a maximum longwave of 410 ± 4 nm, typical for quercetin 3-*O*-glycosides [60] (Figure 5).

**Figure 6:**
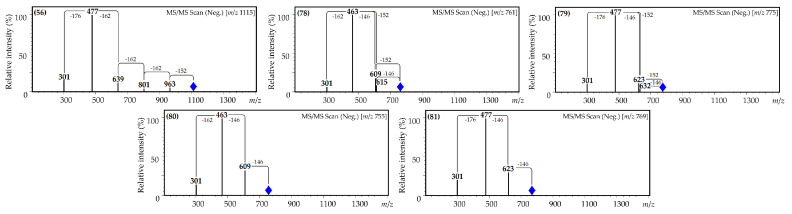
Mass spectra (MS^2^) of acylated quercetin glycosides **56** and **78**–**81**.

The UV spectrum of glycoside **56** was similar to the UV spectrum of non-acylated miquelianin (**68**), but with a hyperchromed band II (Figure 5). The mass spectral primary loss of the fragment with *m*/*z* 152 (*m*/*z* 1115 [M-H]^−^→963) signaled the presence of a galloyl substituent influence on the UV pattern of **56**. The additional removal of three hexose (*m*/*z* 963 [M-H-galloyl]^−^→801, 639 and 477) and one hexuronic moieties (*m*/*z* 477→301) pointed to the structure probably being quercetin 3-*O*-(*O*-galloyl-tri-*O*-hexosyl)-hexuronoside (Figure 6).

The absorption spectra of compounds **78** and **79** included the broad bands I (350 nm) and II (262 nm), and a new middle band at 305 nm, common to coumaroyl esters. In the mass spectra of **78** and **79**, the loss of fragments with *m*/*z* 152 and 146 was registered (*m*/*z* 761 [M-H]^−^→615, 609 for **78**; 775 [M-H]^−^→632, 623 for **79**), indicating the presence of both substituents. The final identification gave the structures of **78** and **79** as quercetin 3-*O*-(*O*-*p*-coumaroyl-*O*-galloyl)-hexoside and quercetin 3-*O*-(*O*-*p*-coumaroyl-*O*-galloyl)-hexuronoside, respectively.

The spectral behavior of compounds **80** and **81** was similar to mono-coumaroylated quercetin glycoside helichrysoside (**73**), but the UV spectral bands at 310 nm were more intensive, as is the case for di-coumaroyl esters [62]. This was confirmed by the mass spectral loss of two coumaroyl fragments, indicating the probable structures of these flavonoids as being quercetin 3-*O*-(di-*O*-*p*-coumaroyl)-hexoside (**80**) and quercetin 3-*O*-(di-*O*-*p*-coumaroyl)-hexuronoside (**81**).

Compounds **65**, **68** and **72** had already been described earlier in *R. canina* and *R. rugosa* [2], and the tentatively identified quercetins have the only analogue of **80** among the known flavonoids: quercetin 3-(3″,6″-di-*O*-*p*-coumaroyl)-glucoside from *Pinus sylvestris* (Pinaceae) [62].

In addition to flavonols, five non-acylated dihydroflavonol glycosides with a specific UV pattern (λ_max_ 290 ± 2 nm) and dihydroquercetin (taxifolin) fragment in mass spectrum (*m*/*z* 303) were identified as dihydroquercetin di-*O*-hexuronide-tri-*O*-hexoside (**51**), *O*-hexuronide-di-*O*-hexoside (**53**), *O*-hexuronide-*O*-hexoside (**55**), *O*-hexoside (**63**) and *O*-hexuronide (**64**). Compound **63** may be the known *O*-galactoside or *O*-glucoside of taxifolin, with substitution of the 3, 5, 7, 3′ or 4′ position of aglycone [63]. The possible analogues of other taxifolins are unknown.

The basic flavonoids of *R. acicularis* leaves were quercetin-3-*O*-glucuronide (**68**), kaempferol-3-*O*-glucuronide (**71**) and kaempferol-*O*-hexuronide-*O*-pentoside (**70**), which had a seasonal content variation of 10.63–21.16 mg/g, 7.03–15.20 mg/g and 3.12–10.65 mg/g, respectively, with a maximal level in July samples for all quantified flavonoids. The total flavonoid content in *R. acicularis* leaves changed from 15.35 mg/g in May to 40.51 mg/g in July and finally to 17.52 mg/g in September. This pattern of change in flavonoid accumulation was previously revealed in other rosaceous plants, such as *Agrimonia asiatica* [23] and *Rubus matsumuranus* [64].

Catechin (**11**), epicatechin (**15**) and epigallocatechin (**9**) were detected in *R. acicularis* leaves using reference standards, and two types of catechin oligomers were found, epicatechin/catechin trimers (**34**, **35**, **37** and **38**) and tetramers (**40**–**42**, **45**). Compounds **11** and **15** were described earlier in *R. canina* and *R. sempervirens* [2], as were di-, tri- and tetrameric catechins in *R. rugosa* and *R. canina* [2]. The maximal seasonal level of catechins and catechin oligomers was revealed as 25.04 mg/g and 4.35 mg/g, respectively, in July. Additionally, 2-pyrone-4,6-dicarboxylic acid (**8**) and three hydroxycinnamates—1-*O*-caffeoylquinic acid (**3**), 5-*O*-caffeoylquinic acid (**10**) and caffeic acid *O*-hexoside (**7**)—were the components of *R. acicularis* leaves in all seasonal stages.

#### 2.2.2. Flowers

**Figure 7:**
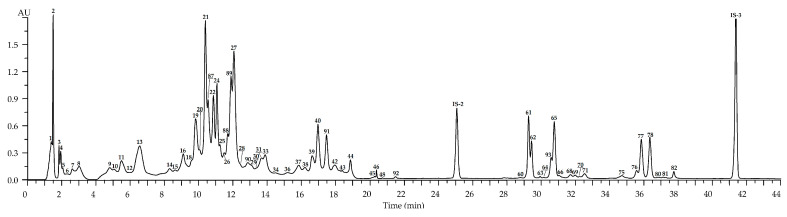
HPLC-PDA chromatogram (270 nm) of *R. acicularis* flower extract. Compounds are numbered as listed in Table 2. Internal standards (IS): IS-2—isovitexin-7,2″-di-*O*-glucoside (25 μg/mL); IS-3—isojaceoside (15 μg/mL).

In total, 67 compounds were identified in *R. acicularis* flower samples (Figure 7, Table 2), most of which were previously described in leaves. Ellagic acid (**29**), known ellagic acid derivatives **6**, **28**, **30**, **31**, **36**, **39**, **43** and **44** and ellagic acid-*O*-hexoside (**90)** were only found in trace amounts or as low-content compounds in flowers, with a total concentration of 4.61 mg/g. Flower ellagitannins included tellimagrandin I_1_ (**14**), I_2_ (**19**), II (**21**), rugosin A (**26**), B_1_ (**16**), D (**27**), E_1_ (**24**), E_2_ (**25**) and three compounds, **87**, **88** and **89,** isomeric to tellimagrandin II, rugosin A and rugosin D, respectively. The principal ellagitannin of *R. acicularis* flowers was tellimagrandin II, which showed a concentration level of 6.03 mg/g, and the total ellagitannin content in flowers was 24.10 mg/g, which was close to the ellagitannin content in leaves collected in May and slightly lower than the July and September leaves. Gallic acid (**2**) and gallotannins **1**, **12**, **13**, **18**, **20**, **33**, **46**, **48** and **92** were also found in *R. acicularis* flowers, and gave a total content of 9.42 mg/g.

Flavonoids, as the main phenolic group in *R. acicularis* flowers, with a total level of 66.72 mg/g, were basically the quercetin (43.43 mg/g) and kaempferol derivatives (20.08 mg/g), with a trace content of dihydroquercetin glycosides. The known quercetin glycosides found in flowers were quercetin-3-*O*-rutinoside (rutin; **65**) and quercetin-3-*O*-glucuronide (miquelianin; **68**), and compounds with possible structures were quercetin *O*-hexuronide-di-*O*-hexoside **61**, quercetin *O*-hexuronide-*O*-hexoside **66**, quercetin *O*-galloyl-*O*-*p*-coumaroyl-*O*-hexoside **78**, quercetin di-*O*-*p*-coumaroyl-*O*-hexoside **80** and quercetin di-*O*-*p*-coumaroyl-*O*-hexuronide **81**. Compounds **61** (16.78 mg/g), **65** (15.25 mg/g) and **78** (11.19 mg/g) amounted to over 60% of the total flavonoid content. Seven kaempferol derivatives included kaempferol-3-*O*-glucuronoide (**71**), kaempferol-3-*O*-(6″-*O*-*p*-coumaroyl)-glucoside (tiliroside; **75**), kaempferol-3-*O*-(6″-*O*-galloyl)-glucoside (**76**), kaempferol di-*O*-hexuronide-di-*O*-hexoside **60**, kaempferol *O*-hexuronide-*O*-hexoside **69**, kaempferol *O*-hexuronide-*O*-pentoside **70** and kaempferol *O*-galloyl-*O*-hexuronide **77**. The acylated flavonoid **77** was the main kaempferol derivative (11.19 mg/g) of *R. acicularis* flower samples, while peonidin 3,5-di-*O*-glucoside (**93**) was the main anthocyanin in *R. acicularis* flowers (3.21 mg/g) and other rose species, such as *R. rugosa* [48], *R. canina* and *R. chinensis* [65].

Three monomeric catechins (**9**, **11** and **15**) and seven catechin oligomers (**34**, **37**, **38**, **40**, **42**, **45** and **91**) gave total contents in flowers of 23.99 mg/g and 18.07 mg/g, respectively. The basic compounds were catechin (19.26 mg/g) and epicatechin/catechin tetramers **40** (8.76 mg/g) and **91** (7.53 mg/g). Mention should also be made of the presence of 2-pyrone-4,6-dicarboxylic acid (**8**) and traces of hydroxycinnamates **3**, **7** and **10** in *R. acicularis* flowers.

#### 2.2.3. Roots

**Figure 8:**
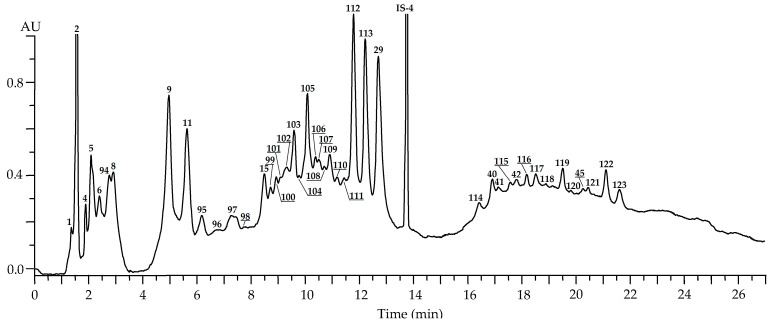
HPLC-PDA chromatogram (270 nm) of *R. acicularis* root extract. Compounds are numbered as listed in Table 2. Internal standard: IS-4—6-*O*-sinapoyl catalpol (30 μg/mL).

The root samples of *R. acicularis* showed no presence of hydroxycinnamates or flavonoids, but high levels of catechins (46.04 mg/g), catechin oligomers (21.80 mg/g) and derivatives of ellagic acid (12.52 mg/g) (Figure 8, Table 2).

Epigallocatechin (**9**; 20.45 mg/g), catechin (**11**; 17.22 mg/g), epicatechin (**15**; 8.23 mg/g) and epicatechin gallate (**111**; 0.14 mg/g) were found in all samples of *R. acicularis* roots, together with numerous catechin oligomers. Seven identified catechin dimers were procyanidin B_1_ (**98**), B_2_ (**105**), B_3_ (**97**) and B_4_ (**100**) as well as their gallic acid esters, procyanidin B_2_ 3-*O*-gallate (**106**), procyanidin B_2_ 3″-*O*-gallate (**107**) and procyanidin B_2_ 3,3″-di-*O*-gallate (**109**). The tentative identification of the remaining compounds showed their nature, as epicatechin/catechin tetramers gave an [M-H]^−^ ion with 1153 a.m.u. (**40**–**42**, **45** and **114**–**117**) and pentamers gave an [M-H]^−^ ion with 1441 a.m.u. (**118**–**123**). Additional *O*-hexosides of catechin dimers **95**, **96**, **99** and **101**–**104,** demonstrating the characteristic loss of a hexose fragment with *m*/*z* 162, were found in *R. acicularis* roots, as well as the epicatechin/catechin dimer *O*-gallate **108,** isomeric to **106** and/or **107**. The basic catechin oligomer was dimeric procyanidin B_2,_ which showed a content level of 7.62 mg/g, and the total content of catechin oligomers in roots of *R. acicularis* was more than in other organs, at about 21.80 mg/g. The presence of dimeric and trimeric procyanidins in the *Rosa* species was demonstrated in *R. rugosa* [66], *R. canina, R. glutinosa, R. rubiginosa, R. multiflora* and *R. spinosissima* [67], but teramers and pentamers were found in the genus for the first time. Ellagic acid (**29**), ellagic acid 4-*O*-rhamnoside (escheweilenol C; **113**) and preliminarily identified ellagic acid tri-*O*-hexoside (**6**) as well as ellagic acid di-*O*-desoxyhexoside (**112**) were found in *R. acicularis* roots, along with two galloyl-hexahydroxydiphenoyl-di-*O*-hexosides, **4** and **5,** and 2-pyrone-4,6-dicarboxylic acid (**8**).

#### 2.2.4. Fruits

**Figure 9:**
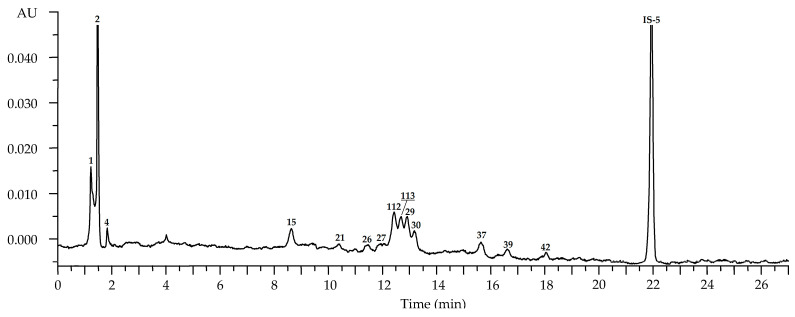
HPLC-PDA chromatogram (270 nm) of *R. acicularis* fruit extract. Compounds are numbered as listed in Table 2. Internal standard: IS-5—neomangiferin (25 μg/mL).

The fruit samples of *R. acicularis* were poor in phenolics, as confirmed by HPLC-PDA-ESI-tQ-MS/MS data (Figure 9, Table 2). Only 14 metabolites were identified, among them the known compounds glucogallin (**1**), gallic acid (**2**), epicatechin (**15**), tellimagrandin II (**21**), rugosin A (**26**), rugosin D (**27**), ellagic acid (**29**) and eschweilenol C (**113**), and six compounds with tentative structures of galloyl-hexahydroxydiphenoyl-di-*O*-hexoside (**4**), ellagic acid methyl ester *O*-hexoside (**30**), epicatechin/catechin trimer (**37**) and tetramer (**42**), di-ellagoyl methyl ester *O*-hexoside (**39**) and ellagic acid di-*O*-desoxyhexoside (**112**). The quantifiable phenolics of *R. acicularis* fruits were gallic acid (1.03 mg/g), eschweilenol C (0.52 mg/g), ellagic acid di-*O*-desoxyhexoside **112** (0.40 mg/g), ellagic acid (0.38 mg/g), epicatechin (0.20 mg/g) and ellagic acid methyl ester *O*-hexoside **30** (0.08 mg/g). The remaining compounds were in very low or trace amounts.

The early study of rose plants showed a wide variation in the contents of fruits with regard to basic phenolics, such as gallic acid in *R. canina* (0.02–0.14 mg/g) [51], *R. rugosa* (0.03–0.85 mg/g) [68], *R. dumalis* in addition to *R. hirtissima* (7.70–12.93 μg/g) [69] and *R. arvensis* (47.9–170.0 μg/g) [70], catechins in *R. canina* in addition to *R. hirtissima* (7.18–50.46 μg/g) [69] and *R. arvensis* (10.4–61.5 μg/g) [70], procyanidins in *R. canina* in addition to *R. dumalis* (7.54–54.41 μg/g) [69] and ellagitannins in *R. canina* (0.43–1.26 mg/g) [51]. The phenolic composition of *R. acicularis* fruits has advantages compared to well-known species such as *R. canina*, *R. rugosa* and others, with their greater phenolic content.

This study demonstrated that the whole *R. acicularis* plant is characterized by organ-specific accumulation of phenolic metabolites: in particular, the basic compounds in the leaves were ellagitannins and gallotannins, while flavonoids dominated in flowers and catechins, monomers and oligomers were found in roots. The lowest content and diversity of phenolics were in the fruits. This can influence the bioactivity of *R. acicularis* extracts as possible inhibitors of digestive enzymes, because variation in the activity of different phenolic groups is known [71,72], as well as the influence of different *Rosa* extracts, based on the α-glucosidase, such as *R. damascena* flowers [73], *R. canina* fruits [74], *R. roxburghii* and *R. sterilis* fruits [75] and *R. acicularis* leaves [23], and on the amylase, such as *R. canina* fruits and flowers [76]. In that regard, it is reasonable to study the interaction with digestive enzymes of extracts from *R. acicularis* organs and define the inhibiting principles of the most active extract.

### 2.3. Digestive-Enzyme-Inhibiting Potential of R. acicularis Extracts and Rugosin D

To study the inhibitory potential of *R. acicularis* extracts on the digestive enzymes, we used the standardized static, simulated gastrointestinal digestion method of Minekus et al., based on physiologically relevant conditions of digestion [77]. In brief, the artificial substrate mixture, including ethylidene-*p*-nitrophenyl-α-D-maltoheptaoside (as a carbohydrate model), *N*_α_-benzoyl-L-arginine-7-amino-4-methylcoumarin hydrochloride (as a protein model) and 4-methylumbelliferyl heptanoate (as a lipid model), was digested with extracts of *R. acicularis* leaves, flowers, roots and fruits (in doses of 100 μg/mL and 1000 μg/mL) by gastric and intestinal enzymatic and electrolyte mixtures (or juices). The gastric phase enzyme was pepsin, and the intestinal phase enzymes included pancreatic amylase, trypsin, chymotrypsin, pancreatic lipase and pancreatic colipase, which allowed for better simulation of physiological digestion enzyme diversity. In a final step, concentrations of specific markers, such as *p*-nitrophenol, 7-amino-4-methylcoumarin and 4-methylumbelliferone, released from an artificial nutrient mixture, were analyzed by an HPLC-DAD assay. The low level of markers indicated the low enzymatic activity of gastrointestinal juices and the high enzyme-inhibiting activity of the studied sample.

**Figure 10:**
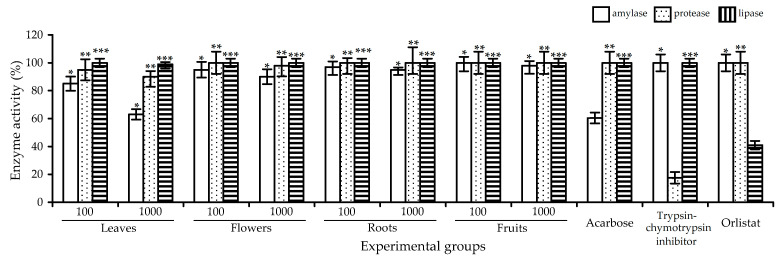
Enzyme activity (percentage of initial) in digestive medium after simulated gastrointestinal digestion of artificial substrate mixture in the presence of *R. acicularis* extracts of leaves, flowers, roots and fruits (100 and 1000 μg/mL), acarbose (1000 μg/mL), trypsin-chymotrypsin inhibitor (1000 μg/mL) and orlistat (1000 μg/mL). *—*p* < 0.05 vs. acarbose group; **—*p* < 0.05 vs. trypsin-chymotrypsin inhibitor group; and ***—*p* < 0.05 vs. orlistat group.

The reference standard enzyme inhibitors demonstrated a high potential against amylase (acarbose, 1000 μg/mL), proteases (trypsin-chymotrypsin inhibitor from *Glycine max*, 1000 μg/mL) and lipases (orlistat, 1000 μg/mL), reducing the initial enzyme activity to 55%, 12% and 38%, respectively (Figure 10). The extract of *R. acicularis* leaves showed a significant reduction in amylase activity, to 84% at the 100 μg/mL dose and to 61% at the 1000 μg/mL dose, with a minor impact on the protease and lipase activity. The extracts of *R. acicularis* flowers, roots and fruits showed little or no influence on the activity of the digestive enzymes. This means that only *R. acicularis* leaf extract possessed a notable inhibitory potential on the amylase and needs further investigation of its active principles.

**Figure 11:**
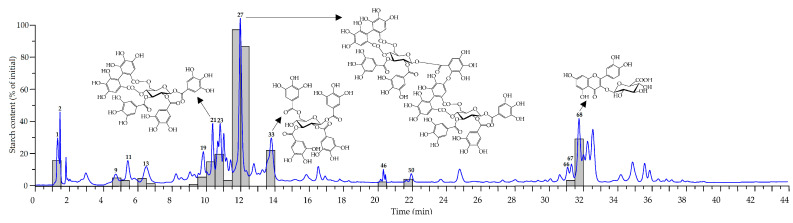
Pancreatic-α-amylase-inhibiting activity of HPLC fraction of *R. acicularis* leaf extract. The grey bars show the starch content (as percentage of initial) in the probe after reaction with pancreatic amylase. The HPLC-PDA profile of *R. acicularis* leaf extract is a blue chromatogram with active compounds numbered as **1**—glucogallin; **2**—gallic acid; **9**—epigallocatechin; **11**—catechin; **13**—1,6-di-*O*-galloyl glucose; **19**—tellimagrandin I_2_; **21**—tellimagrandin II; **23**—tellimagrandin II isomer; **27**—rugosin D; **33**—1,2,3,6-tetra-*O*-galloyl glucose; **46**—penta-*O*-galloyl hexose; **50**—hexa-*O*-galloyl hexose; **66**—quercetin *O*-hexuronoside-*O*-hexoside; **67**—quercetin *O*-hexuronoside-*O*-pentoside; and **68**—miquelianin.

To find the most active metabolite of the *R. acicularis* leaf extract, the HPLC microfractionation technique was applied. The probe of the extract was separated by HPLC, and fractions were eluted every 30 sec, collected, dried and mixed with pancreatic α-amylase. The hydrolytic activity of amylase was studied spectrophotometrically using starch azure as a substrate [78]. Some chromatographic zones demonstrated different effectiveness to protect starch against destructive enzyme influence (Figure 11). The most active was the zone of rugosin D (**27**), which was capable of protecting 82–95% of starch, and medium activity was found for miquelianin (**68**), 1,2,3,6-tetra-*O*-galloyl glucose (**33**), tellimagrandin II (**21**) and tellimagrandin II isomer (**23**).

The dimeric valoneoyl ellagitannin rugosin D and other phenolics were studied previously as *Rosa* *gallica* metabolites with inhibitory activity against bacterial α-amylase from *Bacillus* sp. and fungal α-glucosidase from *Saccharomyces* sp. [78]. The inhibition of rugosin D on mammalian amylases was not found previously, so we have therefore studied the impact of ellagitannin on porcine pancreas α-amylase, human saliva α-amylase and human pancreas α-amylase (Table 3).

The inhibitory potential of rugosin D (IC_50_ 32.09 μg/mL) was comparable to the reference inhibitor acarbose against porcine pancreas α-amylase (IC_50_ 35.67 μg/mL) and exceeded the acarbose activity against both human α-amylases, with the most sensitive to rugosin D being human pancreas α-amylase (IC_50_ 30.84 μg/mL). This was an indication that the bulk ellagitannins of the *Rosa* genus are effective inhibitors of digestive amylases.

**Figure 12:**
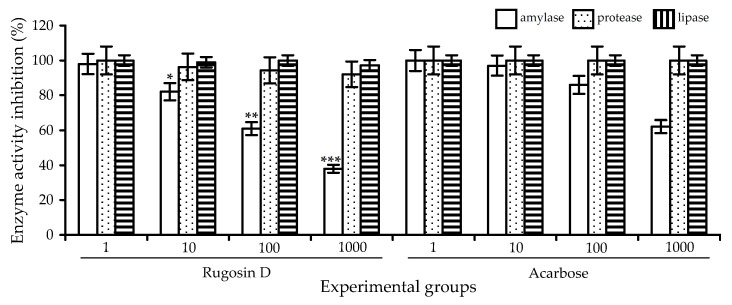
Enzyme activity inhibition (percentage of initial) in digestive fluid after simulated gastrointestinal digestion of artificial substrate mixture in presence of rugosin D (1, 10, 100 and 1000 μg/mL) and acarbose (1, 10, 100 and 1000 μg/mL). * —*p* < 0.05 vs. acarbose group 10 μg/mL; ** —*p* < 0.05 vs. acarbose group 100 μg/mL; and *** —*p* < 0.05 vs. acarbose group 1000 μg/mL.

The simulation of digestion processes with an artificial substrate mixture showed the high effectiveness of rugosin D as an amylase inhibitor, in a dose-dependent manner (Figure 12). The presence of ellagitannin in digestive fluid in doses of 1–1000 μg/mL resulted in 92–38% suppression of amylase activity, while the acarbose demonstrated 99–58% suppression in the same concentration range. The activity of other enzymes, such as proteases and lipases, were not substantially inhibited, indicating the possible selective impact of rugosin on digestive enzymes.

The plant-supporting therapy of diabetes is commonly based on ethnopharmacological data of the application of some extracts, such as hypoglycaemic remedies in traditional medicines [79,80,81]. Among flora, plants of the *Rose* genus are famous antidiabetic medicines with an inhibitory influence on digestive enzymes (α-glucosidase, α-amylase), including *R. canina* [74,78], *R. damascena* [73], *R. gallica* [78], *R. roxburghii* and *R. sterilis* [75]. The prickly rose (*Rosa* *acicularis*) is no exception, and is used in Tibetan and Siberian traditional medicines to prepare antidiabetic decoctions, extracts and tablets [12,13], although it is still an underestimated plant with poor scientific knowledge in regard to its metabolites and bioactivity. In our study of *R. acicularis* organs, many phytochemical classes of a phenolic and non-phenolic nature were quantified in the leaves, flowers, roots and fruits, and phenolic compounds were the most substantive. Use of the HPLC-PDA-ESI-tQ-MS/MS technique allowed for the identification of 123 phenolic compounds in *R. acicularis*, belonging to ellagic acid derivatives, ellagitannins, gallotannins, catechins, catechin oligomers, hydroxycinnamates and flavonoids. The combination of chromatographic and spectrometric data uncovered the variety of new flavonol glycosides, non-acylated and acylated with fragments of gallic acid and *p*-coumaric acids, as well as unknown galloyl hexoses, epicatechin/catechin tri-, tetra- and pentamers. The main phenolics of the leaves were ellagitannins (26.99–70.04 mg/g) and gallotannins (10.80–30.10 mg/g), whilst we found a high concentration of flavonoids (70.72 mg/g) in flowers; catechins and catechin oligomers accumulated in the roots (23.99 mg/g and 18.07 mg/g, respectively). The general metabolic profile of *R. acicularis* was typical for *Rose* plants. The early study of roses found ellagic acid and its glycosides [2], various ellagitannins, such as tellimagrandins in *R. laevigata*, *R. multiflora* and *R. rugosa* [50], rugosins A, B, D and E in *R. canina* [51] and *R. gallica* [78], gallic acid and gallotannins in *R. gallica* [78] and *R. rugosa* [82], flavonoids of kaempferol and quercetin groups in *R. canina*, *R. glauca*, *R. rubiginosa* and *R. sempervirens* [55] as well as dihydroflavonols of the taxifolin group in *R. rugosa* and *R. canina* [2]. While extensive work has been carried out studying prickly rose phenolics, data about other phytochemicals, such as terpenoids, carbohydrates and primary metabolites are not yet complete, and additional chemical and chromatographic studies are need.

The great phenolic diversity of *R. acicularis* implies a wide spectrum of bioactivity, including antidiabetic properties, as in the majority of ellagitannins, flavonoids and gallic acid derivatives [76,77,78]. The model of simulated digestion applied to the artificial mixture demonstrated the specific protection of the carbohydrate substrate against the destructive influence of α-amylase by *R. acicularis* leaf extract. The HPLC fractions of *R. acicularis* leaves containing rugosin D were the most active inhibitors of pancreatic α-amylase activity, and experiments with the pure compound later confirmed the inactivating properties of rugosin D on mammalian α-amylases, comparable with the potency of the known α-amylase inhibitor acarbose. The inhibitory action of mono- and oligomeric ellagitannins of the Rosaceae family on various α-amylases have already been revealed. Tellimagrandin I and II, rugosins A and D as well as casuarictin from *R. gallica* petals were the main inhibitors of α-amylase from *Bacillus stearothermophilus* [78], and rubusuaviins A–F from *Rubus suavissimus* leaves inhibited human salivary α-amylase [83]. Strawberry extracts rich in ellagitannins with different degrees of polymerization demonstrated a pancreatic α-amylase inhibitory activity by decreased postprandial glycaemia of rats [84]. The possible mechanism of α-amylase inactivation may involve the ellagitannin binding of a protein molecule of the digestive enzymes and the formation of insoluble complexes, eventually leading to a reduction in α-amylase activity and the inability to hydrolyze carbohydrate substrates into simple molecules. Rigorous proof of this theory has not been reported in the scientific literature, but the known data about protein–tannin interaction signifies the possibility of protein precipitation by various tannins [85,86]. The protein molecule of α-amylases may precipitate after contact with ellagitannins and inactivation, but of course this fact needs to be proven experimentally. As ellagitannins are often found in the Rosaceae family [50], it can be assumed that the ethnopharmacological evidence of the use of these plants in diabetes is associated with the ability of their extracts to inactivate digestive enzymes by binding to insoluble complexes. The widely distributed rosaceous species *R. acicularis* is an appropriate source of the α-amylase-inactivating ellagitannin rugosin D, and may be a prospective antidiabetic plant for use in the medical and food industry. Our results suggest that future efforts should be focused on the study of ellagitannin–α-amylase interaction to better understand the nature of the antidiabetic potential of this plant extract.

## 3. Materials and Methods

### 3.1. Plant Material and Chemicals

Samples of *Rosa acicularis* (334 samples in total) were collected in the model population in Republic Sakha Yakutia, Lena River left bay, Khangalasskii Ulus, Elanka village (61°17′33.5″ N 128°00′53.8″ E, 720 m a.s.l.; plant density 50–54 plant/100 m^2^) in the pre-flowering period (25–30.V.2020; leaf samples, *n* = 35), the flowering period (15–20.VI.2020; leaf samples, *n* = 81; flower samples, *n* = 45), the fruiting period (28–29.VIII.2020; fruit samples, *n* = 104) and the post-fruiting period (18–22.IX.2020; leaf samples, *n* = 57; root samples, *n* = 12). One sample consisted of 10–15 leaves, 5–7 flowers, 2–3 roots and 30–40 fruits collected from one bush. The species was authenticated by Dr. N.I. Kashchenko (IGEB SB RAS, Ulan-Ude, Russia). For desiccation of the plant material, a ventilated heat oven was used (40 °C, 10–20 days) and dry plants were stored at 3–4 °C before manipulation. The voucher specimens were deposited in the herbarium of the Laboratory of Medical and Biological Research, Institute of General and Experimental Biology (Ulan-Ude, Russia. Specimen No. YA/ROS-0520/14-12–YA/ROS-0520/14-19, YA/ROS-0620/18-24–YA/ROS-0620/18-33, YA/ROS-0820/23-15–YA/ROS-0820/23-22 and YA/ROS-0920/09-14–YA/ROS-0920/09-18).

The reference compounds were purchased from ALB Materials Inc. (Henderson, NV, USA); BioBioPha (Kunming, Yunnan, China); BOC Sciences (Shirley, NY, USA); Carbosynth Ltd. (Compton, Great Britain, UK); ChemFaces (Wuhan, Hubei, China); Extrasynthese (Lyon, France); MCE Med Chem Express (Monmouth, NJ, USA); Sigma-Aldrich (St. Louis, MO, USA); Toronto Research Chemicals (North York, ON, Canada); TransMIT GmbH (Gießen, Germany); or isolated in our laboratory [87,88,89,90,91,92,93,94] (Table S1). The enzymes used in study were α-amylase from porcine pancreas (PMSF-treated, type I-A, saline suspension, ≥1000 units/mg protein; Cat. No. 6255; SigmaAldrich); α-amylase from human saliva (type IX-A, lyophilized powder, 1000–3000 units/mg protein; Cat. No. 0521; Sigma-Aldrich); α-amylase from human pancreas (>400 units/mL; Cat. No. 120-15; Lee Biosolutions, Inc., Maryland Heights, MO, USA); trypsin from bovine pancreas (≥10,000 units/mg protein; Cat. No. T1426; Sigma-Aldrich, St. Louis, MO, USA); α-chymotrypsin from bovine pancreas (type II; ≥40 units/mg protein; Cat. No. C4129; Sigma-Aldrich); lipase from porcine pancreas (type II; ≥650 units/mg protein; Cat. No. L3126; Sigma-Aldrich); and colipase from porcine pancreas (Cat. No. C3028; Sigma-Aldrich). Artificial substrates were ethylidene-*p*-nitrophenyl-α-D-maltoheptaoside (Cat. No. EN45922; Carbosynth Ltd., Compton, Great Britain), *N*_α_-benzoyl-L-arginine-7-amino-4-methylcoumarin hydrochloride (Cat. No. B7260; Sigma-Aldrich) and 4-methylumbelliferyl heptanoate (Cat. No. M2514; Sigma-Aldrich).

### 3.2. Chemical Composition Analysis

Spectrophotometric assays were used for the quantitative analysis of flavonols (as mg/g miquelianin equivalents) [95], dihydroflavonols (as mg/g taxifolin equivalents) [96], catechins (as mg/g (+)-catechin equivalents) [97], hydroxycinnamates (as mg/g 5-*O*-caffeoylquinic acid equivalents) [98], proanthocyanins (as mg/g procyanidin B_1_ equivalents) [99], ellagitannins (as mg/g ellagic acid equivalents) [100], gallotannins (as mg/g gallic acid equivalents) [101], water-soluble polysaccharides (as mg/g glucose equivalents) [102] and carotenoids (as mg/g β-carotene equivalents) [103] by a UV–Vis spectrophotometer, SF-200 (OKB Spectr, Saint Petersburg, Russia), in quartz cells (1 cm). Ascorbic acid was measured by an ascorbic acid assay kit (Cat. No. ab65656; Abcam plc, Cambridge, Great Britain, UK). A titration assay was applied for the quantitative analysis of titratable acids (as mg/g malic acid equivalents) [104], and total lipids were assayed by the Bligh–Dyer method [105]. All the analyses were carried out five times and the data were expressed as mean values ± standard deviation (S.D.).

### 3.3. Plant Extracts Preparation

To prepare the HPLC samples (plant extracts), the ground plant material (particle size 0.125 μm; weight 200 mg) was treated with 70% methanol (2 mL) three times by sonication (ultrasonic bath, 30 min, 45 °C, ultrasound power 100 W, frequency 35 kHz). The liquid extracts were centrifuged (6000× *g*, 10 min) and the supernatant was filtered through 0.22 μm syringe filters into the measuring flask (10 mL). The final volume reached 10 mL with 70% methanol. The liquid methanol extract was stored at 1°C before manipulation. Before analysis, the following internal standards (methanol solutions) were added to samples in a ratio of 1:1: leaf extracts—3′,4′-di-*O*-acetyl-*cis*-khellactone (50 μg/mL); flower extracts—isovitexin-7,2″-di-*O*-glucoside (100 μg/mL) and isojaceoside (60 μg/mL) mixture in a ratio of 1:1; root extracts—6-*O*-sinapoyl catalpol (60 μg/mL); and fruit extracts—neomangiferin (50 μg/mL).

The extracts of *R. acivularis* leaves (July sample), flowers, roots and fruits for the biological study were prepared from the milled samples (60 g), treated by the 70% methanol in an ultrasonic bath (30 min, 45 °C, ultrasound power 100 W, frequency 35 kHz), filtered, reduced in a vacuum until dryness and cold-stored (−20 °C) before analysis. The yields of *R. acivularis* extracts were 32.2% for the leaf sample, 38.4% for the flower sample, 18.61% for the root sample and 32.4% for the fruit sample.

### 3.4. High-Performance Liquid Chromatography with Photodiode Array Detection and Electrospray Ionization Triple Quadrupole Mass Spectrometric Detection (HPLC-PDA-ESI-tQ-MS) Metabolite Profiling

High-performance liquid chromatography with photodiode array detection and electrospray ionization triple quadrupole mass spectrometric detection (HPLC-PDA-ESI-tQ-MS) was used for the quantitative profiling of *R. acicularis* extracts. A liquid chromatograph, LC-20 Prominence, coupled with a photodiode array detector, SPD-M30A (wavelength range of 200–600 nm), triple-quadrupole mass spectrometer, LCMS 8050 (all Shimadzu, Columbia, MD, USA), and GLC Mastro column (2.1 × 150 mm, 3 μm; Shimadzu, Kyoto, Japan) showed better separation of the compounds. The two-component eluent system of eluent A (0.45% CH_3_COOH in water) and B (0.45% CH_3_COOH in MeCN) used the gradient program for the separation: 0–3 min 3%, 3–4 min 3–4%, 4–9 min 4–9%, 9–20 min 9–14%, 20–22 min 14–16%, 22–30 min 16–37%, 33–38 min 37–69%, 38–44 min 69–89% and 44–55 min 89–3%. The injection volume was 1 μL and the flow rate was 120 μL/min. The UV–Vis spectra were recorded by an SPD-M20A photodiode detector (spectral range of 200–600 nm) equipped with a post-column derivatization reactor. The spectra of flavonoid compounds were analyzed before and after the addition of shift reagent (5% AlCl_3_ in acetonitrile). The negative electrospray ionization was used for the mass spectrometric detection of ellagic acid derivatives, ellagitannins, gallotannins, catechins, catechin oligomers, hydroxycinnamates and flavonoid glycosides, and the positive electrospray ionization was used for anthocyanines. The used temperature levels were 300 °C in the ESI interface, 250 °C in the desolvation line and 400 °C in the heat block. The flow values were 3 L/min for the nebulizing gas (N_2_), 10 L/min for the heating gas (air) and 0.3 mL/min for the collision-induced dissociation gas (Ar). The source voltage of mass spectra was 3 kV and the collision energy was −10–35 eV (negative ionization) and +20 (positive ionization) by the scanning range of *m*/*z* 80–1900. The LC-MS system was managed by LabSolution’s workstation software equipped with the inner LC-MS library. The integrated analysis of retention time, ultraviolet and mass spectra data after comparison with the reference standards and literature data was used for the identification of metabolites.

### 3.5. HPLC-ESI-tQ-MS Metabolite Quantification

To quantify 123 metabolites of *R. acicularis* organs, the HPLC-PDA-ESI-tQ-MS conditions used (Section 3.4) and optimized MRM transition MS peak area was used for calculation (Table S2). In total, 34 reference standards were separately weighed (10 mg) and dissolved in the methanol–DMSO mixture (1:1) in volumetric flasks (10 mL) preparing the stock solution (1000 µg/mL) used for the calibration curve building. The calibration solution (1–100 µg/mL) chromatographed in known HPLC-PDA-ESI-tQ-MS conditions and mass spectral data was used to create ‘concentration–mass spectrometric peak area’ correlation. The principal validation criteria, including correlation coefficients (r^2^), standard deviation (S_YX_), limits of detection (LOD), limits of quantification (LOQ) and linear ranges, were found using the known method [27] (Table S3). Five HPLC runs were sufficient for the quantitative analyses, and the results were expressed as mean values ± standard deviation (S.D.).

### 3.6. Simulated Gastrointestinal Digestion

For simulation, the known standardized static gastrointestinal digestion method of Minekus et al. [77] was used with no variation. The artificial substrate mixture was prepared with ethylidene-*p*-nitrophenyl-α-D-maltoheptaoside (0.2% solution in a DMSO–water mixture with a ratio of 2:8), *N*_α_-benzoyl-L-arginine-7-amino-4-methylcoumarin hydrochloride (0.1% solution in a DMSO–methanol–water mixture with a ratio of 1:1:8) and 4-methylumbelliferyl heptanoate (0.1% solution in a DMSO–water mixture with a ratio of 3:7) in a ratio of 1:1:1. An aliquote of the artificial substrate (0.5 mL) was mixed with 0.5 mL of plant extract solution in a DMSO–water mixture (1:9), a pure DMSO–water mixture (1:9; control sample), a standard inhibitor solution (acarbose, trypsin-chymotrypsin inhibitor or orlistat) or rugosin D, 7.5 mL of simulated gastric fluid electrolyte stock solution, 1.6 mL of porcine pepsin solution (25 000 units/mL), 5 mL of CaCl_2_ (0.3 M), 0.2 mL of HCl (1 M) and 0.7 mL of water. The probe was thermostated for 2 h at 37 °C, followed by mixing with 11 mL of simulated intestine fluid electrolyte stock solution, 5.0 mL of an enzyme mixture (porcine trypsin, 100 units/mL; bovine chymotrypsin, 25 units/mL; porcine pancreatic amylase, 200 units/mL; porcine pancreatic lipase, 2000 units/mL; and porcine pancreatic colipase, 1/2 of lipase mass), 2.5 mL of bile (160 mM), 40 mL of CaCl_2_ (0.3 M), 0.15 mL of NaOH (1 M) and 1.31 mL of water, and the probe was then thermostated again for 2 h at 37 °C. Finally, 1 mL of the mixture was mixed with 2 mL of acetonitrile, vortexed (30 s) and then centrifuged (6000× *g*, 10 min). The supernatant was analyzed using an HPLC-DAD assay (Section 3.7) to quantify the level of markers as *p*-nitrophenol, 7-amino-4-methylcoumarin and 4-methylumbelliferone formed from ethylidene-*p*-nitrophenyl-α-D-maltoheptaoside, *N*_α_-benzoyl-L-arginine- 7-amino-4-methylcoumarin hydrochloride and 4-methylumbelliferyl heptanoate, respectively. The level of markers content in the control group (pure DMSO–water mixture with a ratio of 1:9) was 100% of enzyme activity.

### 3.7. Quantification of Gastrointestinal Digestion Markers by HPLC-DAD

Microcolumn high-performance liquid chromatography with diode array detection (HPLC-DAD) was used to separate the markers formed after gastrointestinal digestion of the artificial substrate mixture. The assay was equipped with a MiLiChrom A-02 microcolumn chromatograph (Econova; Novosibirsk, Russia) coupled with a diode array detector and a ProntoSIL-120-5-C18 AQ column (1 × 60 mm, particle diameter 1 μm; Metrohm AG, Herisau, Switzerland). A two-eluent gradient system with 4.1 M of LiClO_4_ in 0.1 M of HCLO_4_ as eluent A and 0.1 M of HCLO_4_ in acetonitrile as eluent B was used to separate samples, and the gradient program was 0–2 min 10–45% B, 2–5 min 45–56% B, 5–10 min 45–100% B and 10–15 min 100–10% B. The parameters of column temperature, injection volume and elution rate were 35 °C, 1 μL and 150 μL/min, respectively. Chromatograms were recorded at 210 nm. The solutions of reference standards (*p*-nitrophenol, 7-amino-4-methylcoumarin and 4-methylumbelliferone) were used to compare the sample chromatograms with a standard chromatogram (Figure S1).

### 3.8. HPLC Microfractionation with Post-Column Pancreatic α-Amylase Inhibition

HPLC-PDA-ESI-tQ-MS conditions (Section 3.4) were used to separate the enlarged volume of *R. acicularis* leaf extract (July sample; 25 μL). The eluates (60 µL) were collected every 30 s by an automated fraction collector, a Shimadzu FRC-10A, and dried under a stream of N_2_. The eluate residue was dissolved in 50% methanol (50 µL) and added to 50 µL of water, 2.5 mL of Remazol-Brilliant-Blue-R-dyed starch (2% suspension in phosphate buffer, pH of 6.8) and 500 μL α-amylase from the human pancreas (0.4 U/mL), incubated for 50 min (37 °C) and the absorbance was measured at 620 nm. The eluates with the most active α-amylase inhibition prevented the blue color formation instead of the inactive eluates, giving a strong coloration. The value of 100% destruction of Remazol-Brilliant-Blue-R-dyed starch (or 0% content of intact starch compound) was measured for the eluate with a retention time of 0.5–1.0 min.

### 3.9. α-Amylase Inhibitory Activity

The α-amylase-inhibiting potential of rugosin D was studied using a microplate spectrophotometric assay [106] with three mammalian amylases, including α-amylase from porcine pancreas, α-amylase from human saliva and α-amylase from human pancreas. Acarbose was a positive control while water was a negative control. The inhibitory activity was measured as IC_50_ (50% inhibition concentration) in μg/mL, estimated graphically after building the ‘concentration–inhibitory percentage’ correlation.

### 3.10. Statistical Analysis

Statistical analyses were performed by one-way analysis of variance, and the significance of the mean difference was determined by Duncan’s multiple range test. Differences at *p* < 0.05 were considered statistically significant. The results are presented as mean values ± standard deviations (S.D.) of some replicates.

## Figures and Tables

**Table 1:** Phytochemical composition of *Rosa* *acicularis* organs, mg/g dry weight (S.D.) ^1,2^.

Phytochemical Group	Leaves (*n* = 53) ^3^	Flowers (*n* = 42) ^3^	Roots (*n* = 6) ^4^	Fruits (*n* = 104) ^5^
Phenolic compounds				
Flavonols	41.48 (3.31)	67.39 (4.98)	0.11 (0.00)	<0.01
Dihydroflavonols	0.52 (0.04)	0.73 (0.06)	<0.01	<0.01
Catechins	27.37 (2.46)	28.86 (2.40)	43.04 (1.24)	0.76 (0.10)
Hydroxycinnamates	1.47 (0.08)	0.20 (0.02)	<0.01	<0.01
Proanthocyanidins	8.22 (0.57)	16.56 (1.82)	26.04 (1.04)	1.05 (0.14)
Anthocyanins	<0.01	5.34 (0.64)	<0.01	<0.01
Ellagitannins	73.69 (6.63)	33.14 (3.64)	12.83 (0.64)	<0.01
Gallotannins	21.23 (2.01)	8.53 (0.51)	3.16 (0.06)	1.22 (0.15)
Total phenolic compounds	173.98	160.75	85.18	3.03
Non-phenolic compounds				
Water-soluble polysaccharides	41.88 (3.35)	65.14 (5.86)	21.76 (0.65)	58.39 (4.08)
Ascorbic acid	5.37 (0.42)	1.14 (0.07)	<0.01	56.12 (6.17)
Titratable acids	8.23 (0.74)	11.27 (1.01)	2.03 (0.08)	38.26 (3.07)
Carotenoids	0.43 (0.01)	<0.01	<0.01	2.33 (0.21)
Lipids	11.07 (1.28)	5.67 (0.45)	2.53 (0.07)	65.12 (7.16)

^1^ All samples were collected in one model population of *R. acicularis* in 2020. ^2^ *n*—number of samples analyzed. ^3^ Collection time: flowering period (15–20.VI.2020). ^4^ Collection time: post-fruiting period, 18–22.IX.2020. ^5^ Collection time: fruiting period, 28–29.VIII.2020.

**Table 2:** Retention times (t_R_) and mass spectral data of compounds **1**–**123** were found in *R. acicularis* leaves, flowers and roots, in addition to their content in the May, July and September samples.

No	t_R_, min	Compound ^a^ (Ref.)	[M-H]^−^, *m*/*z* ^b^	MS/MS, *m*/*z*	Content, mg/g of Dry Plant Weight ± S.D.				
					Leaves: May (*n* = 35) ^c^	Leaves: July (*n* = 81) ^c^	Leaves: September (*n* = 57) ^c^	Flowers: July (*n* = 45) ^c^	Roots: September (*n* = 12) ^c^
**1**	1.26	1-*O*-Galloyl glucose ^S^ (glucogallin) [24]	331	169	0.93 (0.11)	1.58 (0.14)	0.24 (0.01)	0.43 (0.03)	0.23 (0.03)
**2**	1.48	Gallic acid ^S^ [24]	169		1.04 (0.09)	3.68 (0.44)	10.39 (1.45)	2.27 (0.25)	4.27 (0.59)
**3**	1.79	1-*O*-Caffeoylquinic acid ^S^ [38]	353	191, 179, 173 and 135	0.36 (0.02)	0.53 (0.04)	Traces	0.14 (0.01)	n.d.
**4**	1.95	Galloyl-hexahydroxydiphenoyl-di-*O*-hexoside ^L^ [39]	795	633, 481, 463 and 301	Traces	Traces	Traces	0.10 (0.10)	0.29 (0.03)
**5**	2.11	Galloyl-hexahydroxydiphenoyl-di-*O*-hexoside ^L^ [39]	795	633, 481, 463 and 301	Traces	Traces	Traces	Traces	0.37 (0.04)
**6**	2.41	Ellagic acid tri-*O*-hexoside ^L^ [39,40]	787	625, 463 and 301	Traces	Traces	Traces	Traces	1.26 (0.14)
**7**	2.73	Caffeic acid *O*-hexoside ^L^ [38]	341	179, 165	Traces	Traces	Traces	Traces	n.d.
**8**	2.98	2-Pyrone-4,6-dicarboxylic acid ^S^ [41]	183	139, 111	1.44 (0.12)	2.50 (0.34)	1.86 (0.22)	1.43 (0.15)	0.95 (0.11)
**9**	4.71	Epigallocatechin ^S^ [24]	305	179, 137	2.34 (0.22)	5.39 (0.54)	3.77 (0.40)	4.73 (0.42)	20.45 (1.84)
**10**	5.08	5-*O*-Caffeoylquinic acid ^S^ [38]	353	191, 179 and 165	Traces	0.28 (0.03)	Traces	Traces	n.d.
**11**	5.48	Catechin ^S^ [24]	289	205, 137	12.56 (1.01)	18.63 (2.60)	16.24 (1.78)	19.26 (2.11)	17.22 (1.89)
**12**	5.83	Di-*O*-galloyl hexose ^L^ [24]	483	331, 169 and 125	0.04 (0.00)	0.64 (0.81)	Traces	Traces	n.d.
**13**	6.58	1,6-Di-*O*-galloyl glucose ^S^ [24]	483	331, 169 and 125	1.20 (0.14)	4.26 (0.53)	0.92 (0.10)	4.98 (0.45)	n.d.
**14**	8.27	Tellimagrandin I_1_ ^S^ [41,42]	785, 392 *	301	0.12 (0.01)	0.89 (0.09)	0.05 (0.00)	0.09 (0.00)	n.d.
**15**	8.53	Epicatechin ^S^ [24]	289	205, 137	0.89 (0.93)	1.29 (0.09)	0.46 (0.52)	Traces	8.23 (0.74)
**16**	9.08	Rugosin B_1_ ^S^ [41,42]	953, 476 *	785, 597 and 301	0.08 (0.00)	0.40 (0.04)	0.02 (0.00)	0.76 (0.06)	n.d.
**17**	9.38	Tri-*O*-galloyl hexose ^L^ [24]	635	483, 331, 169 and 125	Traces	0.44 (0.05)	Traces	n.d.	n.d.
**18**	9.59	1,3,6-Tri-*O*-galloyl glucose ^S^ [24]	635	483, 331, 169 and 125	Traces	1.29 (0.11)	Traces	Traces	n.d.
**19**	9.82	Tellimagrandin I_2_ ^S^ [41,42]	785, 392 *	301	0.65 (0.04)	2.93 (0.33)	0.59 (0.04)	2.14 (0.19)	n.d.
**20**	10.02	Tri-*O*-galloyl hexose ^L^ [24]	635	483, 331, 169 and 125	0.26 (0.02)	2.35 (0.28)	0.14 (0.01)	Traces	n.d.
**21**	10.48	Tellimagrandin II_1_ ^S^ [41,42]	937, 468 *	301	2.96 (0.34)	8.98 (0.95)	4.22 (0.51)	6.03 (0.58)	n.d.
**22**	10.72	Rugosin B_2_ ^S^ [41,42]	953, 476 *	785, 597 and 301	0.82 (0.08)	3.86 (0.42)	1.59 (0.17)	2.37 (0.19)	n.d.
**23**	10.88	Tellimagrandin II isomer ^S^ [41,42]	937, 468 *	301	2.50 (0.22)	8.29 (0.91)	5.26 (0.73)	n.d.	n.d.
**24**	11.01	Rugosin E_1_ ^S^ [41,42]	860 *	937, 785, 597 and 301	0.73 (0.06)	1.89 (0.26)	0.56 (0.06)	2.85 (0.40)	n.d.
**25**	11.10	Rugosin E_2_ ^S^ [41,42]	860 *	937, 785, 597 and 301	0.50 (0.05)	1.12 (0.14)	0.27 (0.03)	0.39 (0.03)	n.d.
**26**	11.48	Rugosin A ^S^ [41,42]	1105, 552 *	301	0.28 (0.02)	0.53 (0.06)	0.10 (0.01)	0.11 (0.01)	n.d.
**27**	12.03	Rugosin D ^S^ [41,42]	936 *, 623 **	917, 851, 765, 749 and 301	18.35 (1.46)	41.15 (4.93)	32.67 (3.25)	4.06 (0.44)	n.d.
**28**	12.42	1-*O*-Ellagoyl glucose ^S^ [39,40]	463	301	Traces	0.62 (0.05)	Traces	Traces	n.d.
**29**	12.78	Ellagic acid ^S^ [39,40]	301		0.25 (0.03)	2.37 (0.33)	4.29 (0.58)	Traces	4.25 (0.51)
**30**	13.05	Ellagic acid methyl ester *O*-hexoside ^L^ [39,40]	477	315, 301	Traces	0.36 (0.02)	Traces	Traces	n.d.
**31**	13.42	Ellagic acid methyl ester *O*-hexoside ^L^ [39,40]	477	315, 301	Traces	0.45 (0.05)	Traces	0.62 (0.05)	n.d.
**32**	13.52	Tetra-*O*-galloyl hexose ^L^ [24]	787	635, 483, 331 and 169	7.33 (0.87) ^d^	10.54 (1.14) ^d^	4.27 (0.51) ^d^	n.d.	n.d.
**33**	13.82	1,2,3,6-Tetra-*O*-galloyl glucose ^S^ [24]	787	635, 483, 331 and 169				0.97 (0.10)	n.d.
**34**	14.48	Epicatechin/catechin trimer ^L^ [24]	865	577, 451, 407 and 287	Traces	Traces	Traces	Traces	n.d.
**35**	14.67	Epicatechin/catechin trimer ^L^ [24]	865	577, 451, 407 and 287	Traces	Traces	Traces	n.d.	n.d.
**36**	15.21	Ellagic acid dimethyl ester *O*-hexoside ^L^ [39,40]	491	329, 301	Traces	Traces	Traces	Traces	n.d.
**37**	15.82	Epicatechin/catechin trimer ^L^ [24]	865	577, 451, 407 and 287	Traces	2.14 (0.22)	Traces	0.35 (0.02)	n.d.
**38**	16.04	Epicatechin/catechin trimer ^L^ [24]	865	577, 451, 407 and 287	Traces	Traces	Traces	0.29 (0.02)	n.d.
**39**	16.54	Di-ellagoyl methyl ester *O*-hexoside ^L^ [39,40]	761	477, 315 and 301	0.26 (0.03)	0.94 (0.11)	0.14 (0.01)	2.45 (0.29)	n.d.
**40**	16.97	Epicatechin/catechin tetramer ^L^ [24]	1153	863, 577, 575, 451, 407 and 287	Traces	1.95 (0.22)	0.11 (0.01)	8.76 (0.96)	0.73 (0.06)
**41**	17.09	Epicatechin/catechin tetramer ^L^ [24]	1153	863, 577, 451, 407 and 287	Traces	Traces	Traces	n.d.	0.07 (0.01)
**42**	17.81	Epicatechin/catechin tetramer ^L^ [24]	1153	863, 577, 451, 407 and 287	Traces	0.26 (0.02)	Traces	1.14 (0.10)	0.05 (0.00)
**43**	18.42	Di-ellagoyl dimethyl ester *O*-hexoside ^L^ [39,40]	775	477, 315 and 301	Traces	Traces	Traces	Traces	n.d.
**44**	18.80	Ellagic acid trimethyl ester *O*-hexoside ^L^ [39,40]	505	343, 301	Traces	Traces	Traces	1.54 (0.18)	n.d.
**45**	20.14	Epicatechin/catechin tetramer ^L^ [24]	1153	863, 577, 451, 407 and 287	Traces	Traces	Traces	Traces	Traces
**46**	20.38	Penta-*O*-galloyl hexose ^L^ [24]	939	787, 635, 483, 331 and 169	Traces	1.80 (0.22)	Traces	0.77 (0.08)	n.d.
**47**	20.45	1,2,3,4,6-Penta-*O*-galloyl glucose ^S^ [24]	939	787, 635, 483, 331 and 169	Traces	0.53 (0.06)	Traces	n.d.	n.d.
**48**	20.73	Hexa-*O*-galloyl hexose ^L^ [24]	1091	939, 787, 635, 483, 331 and 169	Traces	Traces	Traces	Traces	n.d.
**49**	21.59	Hexa-*O*-galloyl hexose ^L^ [24]	1091	939, 787, 635, 483, 331, 169	Traces	Traces	Traces	n.d.	n.d.
**50**	21.98	Hexa-*O*-galloyl hexose ^L^ [24]	1091	939, 787, 635, 483, 331 and 169	Traces	2.99 (0.33)	0.84 (0.73)	n.d.	n.d.
**51**	23.11	Dihydroquercetin di-*O*-hexuronoside-di-*O*-hexoside ^L^ [43]	979	817, 655, 479 and 303	Traces	Traces	Traces	n.d.	n.d.
**52**	23.76	Quercetin di-*O*-hexuronoside-tri-*O*-hexoside ^L^ [39,44,45]	1139	977, 815, 653, 477 and 301	Traces	0.12 (0.01)	Traces	n.d.	n.d.
**53**	24.27	Dihydroquercetin *O*-hexuronoside-di-*O*-hexoside ^L^ [43]	803	641, 479 and 303	Traces	Traces	Traces	n.d.	n.d.
**54**	24.80	Quercetin *O*-hexuronoside-tri-*O*-hexoside ^L^ [39,44,45]	963	801, 639, 477 and 301	0.64 (0.05)	1.52 (0.12)	0.21 (0.2)	n.d.	n.d.
**55**	25.63	Dihydroquercetin *O*-hexuronoside-*O*-hexoside ^L^ [43]	641	479, 303	Traces	Traces	Traces	n.d.	n.d.
**56**	28.44	Quercetin *O*-hexuronoside-tri-*O*-hexoside-*O*-gallate ^L^ [39,44,45,46]	1115	963, 801, 639, 477 and 301	Traces	Traces	Traces	n.d.	n.d.
**57**	26.78	Quercetin di-*O*-hexuronoside-di-*O*-hexoside ^L^ [39,44,45]	977	815, 653, 477 and 301	Traces	Traces	Traces	n.d.	n.d.
**58**	27.31	Kaempferol *O*-hexuronoside-tri-*O*-hexoside ^L^ [39,44,45]	947	785, 623, 461 and 285	Traces	Traces	Traces	n.d.	n.d.
**59**	28.02	Kaempferol *O*-hexuronoside-tri-*O*-hexoside-*O*-gallate ^L^ [39,44,45,46]	1099	947, 785, 623, 461 and 285	Traces	Traces	Traces	n.d.	n.d.
**60**	28.76	Kaempferol di-*O*-hexuronoside-di-*O*-hexoside ^L^ [39,44,45]	961	799, 637, 461 and 285	Traces	Traces	Traces	Traces	n.d.
**61**	29.01	Quercetin *O*-hexuronoside-di-*O*-hexoside ^L^ [39,44]	801	639, 477 and 301	Traces	Traces	Traces	16.78 (2.01)	n.d.
**62**	29.44	Kaempferol *O*-hexuronoside-di-*O*-hexoside ^L^ [39,44,45]	785	623, 461 and 285	Traces	Traces	Traces	7.37 (0.74)	n.d.
**63**	29.81	Dihydroquercetin *O*-hexoside ^L^ [43]	465	303	Traces	Traces	Traces	Traces	n.d.
**64**	30.10	Dihydroquercetin *O*-hexuronoside ^L^ [43]	479	303	0.22 (0.03)	0.35 (0.03)	0.39 (0.04)	Traces	n.d.
**65**	30.71	Quercetin-*O*-rutinoside ^S^ (rutin) [39,44,45]	609	463, 301	0.10 (0.01)	0.28 (0.03)	0.06 (0.00)	15.25 (1.67)	n.d.
**66**	31.09	Quercetin *O*-hexuronoside-*O*-hexoside ^L^ [39,44,45]	639	477, 301	0.16 (0.01)	0.88 (0.09)	0.12 (0.01)	Traces	n.d.
**67**	31.42	Quercetin *O*-hexuronoside-*O*-pentoside ^L^ [39,44,45]	609	477, 301	0.52 (0.04)	3.04 (0.28)	0.83 (0.09)	n.d.	n.d.
**68**	31.83	Quercetin-3-*O*-glucuronide ^S^ (miquelianin) [24,39,44]	477	301	5.31 (0.42)	10.08 (1.22)	9.35 (0.94)	0.21 (0.02)	n.d.
**69**	32.02	Kaempferol *O*-hexuronoside-*O*-hexoside ^L^ [39,44]	623	461, 285	1.11 (0.09)	3.16 (0.37)	0.51 (0.05)	Traces	n.d.
**70**	32.40	Kaempferol *O*-hexuronoside-*O*-pentoside ^L^ [39,44]	593	461, 285	1.57 (0.12)	5.38 (0.51)	2.21 (0.25)	Traces	n.d.
**71**	32.62	Kaempferol-3-*O*-glucuronide ^S^ [24,39,44]	461	285	3.61 (0.28)	7.63 (0.79)	3.37 (0.34)	0.12 (0.01)	n.d.
**72**	33.24	Quercetin-3-*O*-rhamnoside ^S^ (quercitrin) [24,39,44]	447	301	Traces	Traces	Traces	n.d.	n.d.
**73**	33.76	Quercetin-3-*O*-(6″-*O*-*p*-coumaroyl)-glucoside ^S^ (helichrysoside) [24,39,44]	609	463, 301	Traces	Traces	Traces	n.d.	n.d.
**74**	34.22	Kaempferol-3-*O*-arabinoside ^S^ (juglanin) [24,39,44]	417	285	0.22 (0.02)	0.40 (0.04)	Traces	n.d.	n.d.
**75**	34.97	Kaempferol-3-*O*-(6″-*O*-*p*-coumaroyl)-glucoside ^S^ (tiliroside) [24,39,44]	593	447, 285	1.73 (0.16)	2.14 (0.23)	0.47 (0.04)	0.31 (0.02)	n.d.
**76**	35.63	Kaempferol-3-*O*-(6″-*O*-galloyl)-glucoside ^S^ [39,44,45,46]	599	447, 285	0.12 (0.01)	5.03 (0.52)	Traces	0.98 (0.09)	n.d.
**77**	35.96	Kaempferol *O*-hexuronoside-*O*-gallate ^L^ [39,44,45,46]	613	461, 285	0.04 (0.00)	0.35 (0.03)	Traces	10.95 (1.28)	n.d.
**78**	36.47	Quercetin *O*-hexoside-*O*-*p*-coumarate-*O*-gallate ^L^ [39,44,45,46]	761	615, 609, 463 and 301	Traces	0.15 (0.01)	Traces	11.19 (1.01)	n.d.
**79**	36.69	Quercetin *O*-hexuronoside-*O*-*p*-coumarate-*O*-gallate ^L^ [39,44,45,46,47]	775	632, 623, 477 and 301	Traces	Traces	Traces	n.d.	n.d.
**80**	36.97	Quercetin *O*-hexoside-di-*O*-*p*-coumarate ^L^ [24,39,44,47]	755	609, 463 and 301	Traces	Traces	Traces	Traces	n.d.
**81**	37.15	Quercetin *O*-hexuronoside-di-*O*-*p*-coumarate ^L^ [24,39,44,47]	769	623, 477 and 301	Traces	Traces	Traces	Traces	n.d.
**82**	37.90	Kaempferol *O*-hexuronoside-*O*-*p*-coumarate-*O*-gallate ^L^ [39,44,45,46,47]	759	613, 607, 461 and 285	Traces	Traces	Traces	0.35 (0.03)	n.d.
**83**	38.09	Kaempferol *O*-hexuronoside-*O*-hexoside-di-*O*-*p*-coumarate ^L^ [24,39,44,47]	915	769, 623, 461 and 285	Traces	Traces	Traces	n.d.	n.d.
**84**	38.93	Kaempferol *O*-hexoside-di-*O*-*p*-coumarate ^L^ [24,39,44,47]	739	593, 447 and 285	Traces	Traces	Traces	n.d.	n.d.
**85**	39.05	Kaempferol *O*-hexuronoside-di-*O*-*p*-coumarate ^L^ [24,39,44,47]	753	607, 461 and 285	Traces	Traces	Traces	n.d.	n.d.
**86**	39.58	Kaempferol *O*-hexuronoside-tri-*O*-*p*-coumarate ^L^ [24,39,44,47]	899	753, 607, 461 and 285	Traces	Traces	Traces	n.d.	n.d.
**87**	10.53	Hexahydroxydiphenoyl-tri-*O*-galloyl-hexose (tellimagrandin II isomer) ^L^ [41,42]	937, 468 *	301	n.d.	n.d.	n.d.	1.95 (0.20)	n.d.
**88**	11.80	Valoneoyl-tri-*O*-galloyl-hexose (rugosin A isomer) ^L^ [41,42]	1105, 552 *	301	n.d.	n.d.	n.d.	0.52 (0.04)	n.d.
**89**	11.98	Hexahydroxydiphenoyl-valoneoyl-tetra-*O*-galloyl- di-*O*-hexose (rugosin D isomer) ^L^ [41,42]	936 *, 623 **	917, 851, 765, 749 and 301	n.d.	n.d.	n.d.	2.73 (0.38)	n.d.
**90**	12.81	Ellagic acid *O*-hexoside ^L^ [39,40]	463	301	n.d.	n.d.	n.d.	Traces	n.d.
**91**	17.49	Epicatechin/catechin tetramer ^L^ [24]	1153	863, 577, 575, 451, 407 and 287	n.d.	n.d.	n.d.	7.53 (1.05)	n.d.
**92**	21.50	Hexa-*O*-galloyl hexoside ^L^ [24]	1091	939, 787, 635, 483, 331 and 169	n.d.	n.d.	n.d.	Traces	n.d.
**93**	31.12	Peonidin 3,5-di-*O*-glucoside ^S^ [48]	625 ^e^	463, 301 ^e^	n.d.	n.d.	n.d.	3.21 (0.35)	n.d.
**94**	2.84	Ellagic acid tri-*O*-hexoside ^L^ [39,40]	787	625, 463 and 301	n.d.	n.d.	n.d.	n.d.	1.63 (0.19)
**95**	6.18	Epicatechin/catechin dimer *O*-hexoside ^L^ [24]	739	577, 451, 407 and 287	n.d.	n.d.	n.d.	n.d.	1.14 (0.14)
**96**	6.57	Epicatechin/catechin dimer *O*-hexoside ^L^ [24]	739	577, 451, 407 and 287	n.d.	n.d.	n.d.	n.d.	Traces
**97**	7.29	Procyanidin B_3_^S^ [24]	577	451, 407 and 287	n.d.	n.d.	n.d.	n.d.	1.29 (0.15)
**98**	7.71	Procyanidin B_1_^S^ [24]	577	451, 407 and 287	n.d.	n.d.	n.d.	n.d.	Traces
**99**	8.52	Epicatechin/catechin dimer *O*-hexoside ^L^ [24]	739	577, 451, 407 and 287	n.d.	n.d.	n.d.	n.d.	Traces
**100**	8.82	Procyanidin B_4_^S^ [24]	577	451, 407 and 287	n.d.	n.d.	n.d.	n.d.	0.11 (0.01)
**101**	9.01	Epicatechin/catechin dimer *O*-hexoside ^L^ [24]	739	577, 451, 407 and 287	n.d.	n.d.	n.d.	n.d.	Traces
**102**	9.11	Epicatechin/catechin dimer *O*-hexoside ^L^ [24]	739	577, 451, 407 and 287	n.d.	n.d.	n.d.	n.d.	0.10 (0.01)
**103**	9.47	Epicatechin/catechin dimer *O*-hexoside ^L^ [24]	739	577, 451, 407 and 287	n.d.	n.d.	n.d.	n.d.	3.84 (0.42)
**104**	9.58	Epicatechin/catechin dimer *O*-hexoside ^L^ [24]	739	577, 451, 407 and 287	n.d.	n.d.	n.d.	n.d.	Traces
**105**	10.00	Procyanidin B_2_^S^ [24]	577	451, 407 and 287	n.d.	n.d.	n.d.	n.d.	7.62 (0.91)
**106**	10.25	Procyanidin B_2_ 3-*O*-gallate ^S^ [49]	729	577, 559, 541, 441 and 289	n.d.	n.d.	n.d.	n.d.	Traces
**107**	10.41	Procyanidin B_2_ 3″-*O*-gallate ^S^ [49]	729	577, 559, 541, 441 and 289	n.d.	n.d.	n.d.	n.d.	Traces
**108**	10.52	Epicatechin/catechin dimer *O*-gallate ^L^ [49]	729	577, 541, 441 and 289	n.d.	n.d.	n.d.	n.d.	Traces
**109**	10.78	Procyanidin B_2_ 3,3″-di-*O*-gallate ^S^ [49]	881	729, 577, 559, 541, 441 and 289	n.d.	n.d.	n.d.	n.d.	0.96 (0.10)
**110**	11.09	1-*O*-Ellagoyl-gentiobiose (amritoside) ^S^ [39,40]	625	463, 301	n.d.	n.d.	n.d.	n.d.	Traces
**111**	11.41	Epicatechin gallate ^S^ [24]	441		n.d.	n.d.	n.d.	n.d.	0.14 (0.01)
**112**	11.72	Ellagic acid di-*O*-desoxyhexoside ^L^ [39,40]	593	447, 301	n.d.	n.d.	n.d.	n.d.	3.10 (0.43)
**113**	12.05	Ellagic acid 4-*O*-rhamnoside (eschweilenol C) ^S^ [39,40]	447	301	n.d.	n.d.	n.d.	n.d.	3.91 (0.46)
**114**	16.41	Epicatechin/catechin tetramer ^L^ [24]	1153	863, 577, 575, 451, 407, 287	n.d.	n.d.	n.d.	n.d.	0.31 (0.02)
**115**	17.51	Epicatechin/catechin tetramer ^L^ [24]	1153	863, 577, 575, 451, 407 and 287	n.d.	n.d.	n.d.	n.d.	Traces
**116**	18.10	Epicatechin/catechin tetramer ^L^ [24]	1153	863, 577, 575, 451, 407 and 287	n.d.	n.d.	n.d.	n.d.	0.40 (0.04)
**117**	18.48	Epicatechin/catechin tetramer ^L^ [24]	1153	863, 577, 575, 451, 407 and 287	n.d.	n.d.	n.d.	n.d.	0.46 (0.04)
**118**	18.97	Epicatechin/catechin pentamer ^L^ [24]	1441	1153, 863, 577, 451, 407 and 287	n.d.	n.d.	n.d.	n.d.	Traces
**119**	19.50	Epicatechin/catechin pentamer ^L^ [24]	1441	1153, 863, 577, 451, 407 and 287	n.d.	n.d.	n.d.	n.d.	1.26 (0.17)
**120**	19.72	Epicatechin/catechin pentamer ^L^ [24]	1441	1153, 863, 577, 451, 407 and 287	n.d.	n.d.	n.d.	n.d.	Traces
**121**	20.41	Epicatechin/catechin pentamer ^L^ [24]	1441	1153, 863, 577, 451, 407 and 287	n.d.	n.d.	n.d.	n.d.	Traces
**122**	21.01	Epicatechin/catechin pentamer ^L^ [24]	1441	1153, 863, 577, 451, 407 and 287	n.d.	n.d.	n.d.	n.d.	1.87 (0.26)
**123**	21.52	Epicatechin/catechin pentamer ^L^ [24]	1441	1153, 863, 577, 451, 407 and 287	n.d.	n.d.	n.d.	n.d.	1.59 (0.22)
Total phenolic content,					71.24	178.09	106.41	148.48	88.10
*incl.* ellagic acid and hexosides					0.51	4.74	4.43	4.61	14.15
ellagitannins					26.99	70.04	45.33	24.10	0.66
gallotannins					10.80	30.10	16.80	9.42	4.50
catechins					15.79	25.04	20.47	23.99	46.04
catechin oligomers					Traces	4.35	Traces	18.07	21.80
hydroxycinnamates					0.36	0.81	Traces	0.14	n.d.
flavonoids,					15.35	40.51	17.52	66.72	n.d.
*incl.* quercetin glycosides					6.73	16.07	10.57	43.43	n.d.
kaempferol glycosides					8.40	24.09	6.56	20.08	n.d.
dihydroquercetin glycosides					0.22	0.35	0.39	Traces	n.d.
anthocyanins					n.d.	n.d.	n.d.	3.21	Traces
various compounds					1.44	2.50	1.86	1.43	0.95

^a^ Compound identification was based on the comparison of retention time, UV and MS spectral data with reference standards (^S^) or the interpretation of UV and MS spectral data in comparison with literature data (^L^). ^b^ * [M-2H]^2−^. ** [M-3H]^3−^. ^c^ In brackets—number of samples analyzed. ^d^ Calculated as a sum of compounds **32** and **33**. ^e^ Positive ionization. Abbreviations used: Ara—arabinose; *p*Cou—*p*-coumaroyl; EllA—ellagic acid; Glc—glucose; GlcA—glucuronic acid; Gall—galloyl; Hex—hexose; HexA—hexuronic acid; HHDP—hexahydroxydiphenoyl; Me—methyl; Rha—rhamnose; Rut—rutinose; Val—valoneoyl; n.d.—not detected; and traces—<LOQ (limit of quantification).

**Table 3:** Inhibitory effects of rugosin D and acarbose against mammalian α-amylases, IC_50_ (μg/mL ± S.D.).

Compound	Porcine Pancreas α-Amylase	Human Saliva α-Amylase	Human Pancreas α-Amylase
Rugosin D	32.09 ± 1.21	67.59 ± 4.12 *	30.84 ± 1.23 *
Acarbose	35.67 ± 1.42	82.33 ± 3.54	56.39 ± 2.81

*—*p* < 0.05 vs. acarbose.

## Data Availability

Data are contained within the article.

## Supplementary Materials

The following are available online at https://www.mdpi.com/article/10.3390/plants10112525/s1, Table S1: Reference standards and internal standards used for the qualitative and quantitative analysis by HPLC-MS and HPLC-DAD, Table S2: Optimized MRM transitions of compounds 1–123 in HPLC-MS/MS analysis, Table S3: Regression equations, correlation coefficients, standard deviation, limits of detection, limits of quantification and linear ranges for 34 reference standards and Figure S1: HPLC-DAD chromatograms of gastrointestinal digestion markers after digestion of the artificial substrate mixture.

## References

[1] Bruneau A., Starr J.R., Joly S. (2007). Phylogenetic relationships in the genus *Rosa*: New evidence from chloroplast DNA sequences and an appraisal of current knowledge. Syst. Bot..

[2] Ayati Z., Amiri M.S., Ramezani M., Delshad E., Sahebkar A., Emami S.A. (2018). Phytochemistry, traditional uses and pharmacological profile of rose hip: A review. Curr. Pharm. Des..

[3] Malyschev L.I. (2004). Flora of Siberia.

[4] Sokolov P.D. (1987). Plant Recourses of USSR.

[5] Lewis W.H. (1959). A monograph of the genus *Rosa* in North America. I. R. acicularis. Brittonia.

[6] Shishmareva T.M., Shishmarev V.M. (2017). Resources of Medicinal Plants of Transbaikalia.

[7] Nikitin G.I. (1957). Wild Fruits and Berries of Sakhalin and Kuriles.

[8] Shreter A.I. (1975). The Medical Flora of Soviet Far East.

[9] Makarov A.A. (1989). Bioactive Compounds in Plants of Yakutia.

[10] Makarov A.A. (1974). Plant Medical Remedies of Yakut Traditional Medicine.

[11] Sanchzhai-Chzhamso D., Dashiev D.B.T., Aseeva T.A. (2014). Vaiduria Onbo. The Blue Beryl.

[12] Batorova S.M., Yakovlev G.P., Aseeva T.A. (2013). Reference-Book of Traditional Tibetan Medicine Herb.

[13] Aseeva T.A. (2016). Digestive Diseases: Symptomatology and Treatment (by Materials of Tibetan Medical Books).

[14] Lu J., Wang C. (2018). Medicinal components and pharmacological effects of *Rosa rugosa*. Rec. Nat. Prod..

[15] Patel S. (2017). Rose hip as an underutilized functional food: Evidence-based review. Trends Food Sci. Technol..

[16] Enkhtuya E., Kashiwagi T., Shimamura T., Ukeda H., Tseye-Oidov O. (2014). Screening study on antioxidant activity of plants grown wildly in Mongolia. Food Sci. Technol. Res..

[17] Kobayashi K., Takahashi T., Takano F., Fushiya S., Batkhuu J., Sanchir C., Yoshizaki F. (2004). Survey of the influence of Mongolian plants on lipase activity in mouse plasma and gastrointestinal tube. Nat. Med..

[18] Park J.C., Kim S.C., Choi M.R., Song S.H., Yoo E.J., Kim S.H., Miyashiro H., Hattori M. (2005). Anti-HIV protease activity from *Rosa* family plant extracts and rosamultin from *Rosa rugosa*. J. Med. Food.

[19] Gonchig E., Erdenebat S., Togtoo O., Bataa S., Gendaram O., Young S.K., Shi Y.R. (2008). Antimicrobial activity of Mongolian medicinal plants. Nat. Prod. Sci..

[20] Afanasyeva L.V., Ayushina T.A. (2019). Features of microelements accumulation in *Rosa acicularis* plants. Khimiya Rastitel’nogo Syr’ya.

[21] Zhao M.-Y., Zhao Y.-H. (2019). Analysis and comparison of essential oil composition between *Rosa acicularis* ‘Luhe’ and Lindl. Modern Food Sci. Technol..

[22] Zhao Y.-H., Shi S.-S., Zhang L.-G. (2019). Bioactive compounds extracted by different solvents from *Rosa acicularis* ’Luhe’ leaves and their antioxidant activity. Modern Food Sci. Technol..

[23] Kashchenko N.I., Olennikov D.N. (2020). Phenolome of Asian agrimony tea (*Agrimonia asiatica* Juz., Rosaceae): LC-MS profile, α-glucosidase inhibitory potential and stability. Foods.

[24] Olennikov D.N., Chirikova N.K., Vasilieva A.G., Fedorov I.A. (2020). LC-MS profile, gastrointestinal and gut microbiota stability and antioxidant activity of *Rhodiola rosea* herb metabolites: A comparative study with subterranean organs. Antioxidants.

[25] Olennikov D.N., Kashchenko N.I., Chirikova N.K., Vasil’eva A.G., Gadimli A.I., Isaev J.I., Vennos C. (2019). Caffeoylquinic acids and flavonoids of fringed sagewort (*Artemisia frigida* Willd.): HPLC-DAD-ESI-QQQ-MS profile, HPLC-DAD quantification, *in vitro* digestion stability, and antioxidant capacity. Antioxidants.

[26] Olennikov D.N., Gadimli A.I., Isaev J.I., Kashchenko N.I., Prokopyev A.S., Katayeva T.N., Chirikova N.K., Vennos C. (2019). Caucasian *Gentiana* species: Untargeted LC-MS metabolic profiling, antioxidant and digestive enzyme inhibiting activity of six plants. Metabolites.

[27] Olennikov D.N., Chirikova N.K., Kashchenko N.I., Nikolaev V.M., Kim S.-W., Vennos C. (2018). Bioactive phenolics of the genus *Artemisia* (Asteraceae): HPLC-DAD-ESI-TQ-MS/MS profile of the Siberian species and their inhibitory potential against α-amylase and α-glucosidase. Front. Pharmacol..

[28] Kashchenko N.I., Chirikova N.K., Olennikov D.N. (2018). Acylated flavonoids from *Spiraea* genus as inhibitors of α-amylase. Russ. J. Bioorg. Chem..

[29] Kashchenko N.I., Chirikova N.K., Olennikov D.N. (2017). Agrimoniin, an active ellagitannin from *Comarum palustre* herb with anti-α-glucosidase and antidiabetic potential in streptozotocin-induced diabetic rats. Molecules.

[30] Bitis L., Sen A., Ozsoy N., Birteksoz-Tan S., Kultur S., Melikoglu G. (2017). Flavonoids and biological activities of various extracts from *Rosa sempervirens* leaves. Biotechnol. Biotechnol. Equip..

[31] Nowak R., Gawlik-Dziki U. (2007). Polyphenols of *Rosa,* L. leaves extracts and their radical scavenging activity. Z. Naturforsch. C.

[32] Koczka N., Stefanovits-Bányai É., Ombódi A. (2018). Total polyphenol content and antioxidant capacity of rosehips of some *Rosa* species. Medicines.

[33] Liaudanskas M., Noreikienė I., Zymonė K., Juodytė R., Žvikas V., Janulis V. (2021). Composition and antioxidant activity of phenolic compounds in fruit of the genus *Rosa* L.. Antioxidants.

[34] Olech M., Nowacka-Jechalke N., Masłyk M., Martyna A., Pietrzak W., Kubiński K., Załuski D., Nowak R. (2019). Polysaccharide-rich fractions from *Rosa rugosa* Thunb.—Composition and chemopreventive potential. Molecules.

[35] Oprica L., Bucsa C., Zamfirache M.M. (2015). Ascorbic acid content of rose hip fruit depending on altitude. Iran J. Public Health.

[36] Roman I., Stănilă A., Stănilă S. (2013). Bioactive compounds and antioxidant activity of *Rosa canina* L. biotypes from spontaneous flora of Transylvania. Chem. Centr. J..

[37] Andersson S.C., Rumpunen K., Johansson E., Olsson M.E. (2011). Carotenoid content and composition in rose hips (*Rosa* spp.) during ripening, determination of suitable maturity marker and implications for health promoting food products. Food Chem..

[38] Olennikov D.N., Nikolaev V.M., Chirikova N.K. (2021). Sagan Dalya tea, a new “old” probable adaptogenic drug: Metabolic characterization and bioactivity potentials of *Rhododendron adamsii* leaves. Antioxidants.

[39] Olennikov D.N., Kirillina C.S., Chirikova N.K. (2021). Water-soluble melanoidin pigment as a new antioxidant component of fermented willowherb leaves (*Epilobium angustifolium*). Antioxidants.

[40] Olennikov D.N., Kashchenko N.I., Vennos C. (2019). New ellagic acid glycosides from *Punica granatum*. Chem. Nat. Comp..

[41] Olennikov D.N., Vasilieva A.G., Chirikova N.K. (2020). *Fragaria viridis* fruit metabolites: Variation of LC-MS profile and antioxidant potential during ripening and storage. Pharmaceuticals.

[42] Bijttebier S., Van der Auwera A., Voorspoels S., Noten B., Hermans N., Pieters L., Apers S. (2016). A first step in the quest for the active constituents in *Filipendula ulmaria* (meadowsweet): Comprehensive phytochemical identification by liquid chromatography coupled to quadrupole-orbitrap mass spectrometry. Planta Med..

[43] Yang C.-J., Wang Z.-B., Mi Y.-Y., Gao M.-J., Lv J.-N., Meng Y.-H., Yang B.-Y., Kuang H.-X. (2016). UHPLC-MS/MS determination, pharmacokinetic, and bioavailability study of taxifolin in rat plasma after oral administration of its nanodispersion. Molecules.

[44] Olennikov D.N. (2020). Synanthropic plants as an underestimated source of bioactive phytochemicals: A case of *Galeopsis bifida* (Lamiaceae). Plants.

[45] Wubshet S.G., Moresco H.H., Tahtah Y., Brighente I.M.C., Staerk D. (2015). High-resolution bioactivity profiling combined with HPLC-HRMS-SPE-NMR: α-Glucosidase inhibitors and acetylated ellagic acid rhamnosides from *Myrcia palustris* DC. (Myrtaceae). Phytochemistry.

[46] Masuda Y., Iritani K., Yonemori S., Oyama Y., Takeda Y. (2001). Isolation and antioxidant activity of galloyl flavonol glycosides from the seashore plant. Pemphis Acidula. Biosci. Biotechnol. Biochem..

[47] Skaltsa H., Verykokidou E., Harvala C., Karabourniotis G., Manetasi Y. (1994). UV-B protective potential and flavonoid content of leaf hairs of *Quercus ilex*. Phytochemistry.

[48] Zhao M., Sui X., Wang Y., Xu Z., Yu X., Han X. (2019). Component analysis of anthocyanins in petals at different flowering stages of three *Rosa rugosa* hybrid cultivars. Adv. Biosci. Biotechnol..

[49] Agarwal C., Veluri R., Kaur M., Chou S.-C., Thompson J.A., Agarwal R. (2007). Fractionation of high molecular weight tannins in grape seed extract and identification of procyanidin B2-3,3′-di-*O*-gallate as a major active constituent causing growth inhibition and apoptotic death of DU145 human prostate carcinoma cells. Carcinogenesis.

[50] Okuda T., Yoshida T., Hatano T., Iwasaki M., Kubo M., Orime T., Yoshizaki M., Naruhashi N. (1992). Hydrolysable tannins as chemotaxonomic markers in the *Rosaceae*. Phytochemistry.

[51] Fecka I. (2009). Qualitative and quantitative determination of hydrolysable tannins and other polyphenols in herbal products from meadowsweet and dog rose. Phytochem. Anal..

[52] Cai Y.-Z., Xing J., Sun M., Zhan Z.-Q., Corke H. (2005). Phenolic antioxidants (hydrolyzable tannins, flavonols, and anthocyanins) identified by LC-ESI-MS and MALDI-QIT-TOF MS from *Rosa chinensis* flowers. J. Agric. Food Chem..

[53] Cendrowski A., Scibisz I., Kieliszek M., Kolniak-Ostek J., Mitek M. (2017). UPLC-PDA-Q/TOF-MS Profile of polyphenolic compounds of liqueurs from rose petals (*Rosa rugosa*). Molecules.

[54] Salminen J.-P., Ossipov V., Haukioja E., Pihlaja K. (2001). Seasonal variation in the content of hydrolysable tannins in leaves of *Betula pubescens*. Phytochemistry.

[55] Cunja V., Mikulic-Petkovsek M., Stampar F., Schmitzer V. (2014). Compound identification of selected rose species and cultivars: An insight to petal and leaf phenolic profiles. J. Am. Soc. Hort. Sci..

[56] Budzianowski J. (1991). Six flavonol glucuronides from *Tulipa gesneriana*. Phytochemistry.

[57] Olennikov D.N. (2020). Flavonol glycosides from leaves of *Allium microdictyon*. Chem. Nat. Comp..

[58] Saleh A.M.N. (1985). Flavonol glycosides of *Euphorbia retusa* and *E. sanctae-catharinae*. Phytochemistry.

[59] Urushibara S., Kitayama Y., Watanabe T., Okuno T., Watarai A., Matsumoto T. (1992). New flavonol glycosides, major determinants inducing the green fluorescence in the guard cells of *Allium cepa*. Tetrahedr. Lett..

[60] Markham U.R. (1982). Techniques of Flavonoid Identification.

[61] Zadorozhnii A.M., Zapesochnaya G.G., Pervykh L.N., Shchavlinskii A.N., Kovtun L.S., Svanidze N.V. (1986). Investigation of the herb *Aerva lanata*. 1-*O*-Acylglycosides of flavonoids. Pharm. Chem. J..

[62] Jungblut T.P., Schnitzler J.-P., Heller W., Szymczak W., Metzger J.W. (1995). Structures of UV-B induced sunscreen pigments of the Scots pine (*Pinus sylvestris* L.). Angew. Chem..

[63] Andersen Ø.M., Markham K.R. (2006). Flavonoids: Chemistry, Biochemistry, and Applications.

[64] Kashchenko N.I., Olennikov D.N., Chirikova N.K. (2021). Metabolites of Siberian raspberries: LC-MS profile, seasonal variation, antioxidant activity and thermal stability of *Rubus matsumuranus* phenolome. Plants.

[65] Mikanagi Y., Yokoi M., Ueda Y., Saito N. (1995). Flower flavonol and anthocyanin distribution in subgenus *Rosa*. Biochem. Syst. Ecol..

[66] Park J.C., Ito H., Yoshida T. (2003). ^1^H-NMR assignment of HIV protease inhibitor, procyanidin B3 isolated from *Rosa rugosa*. Nat. Prod. Sci..

[67] Zagorac D.Č.D., Akšić M.M.F., Glavnik V., Gašić U.M., Vovk I., Tešić Ž.L., Natić M.M. (2020). Establishing the chromatographic fingerprints of flavan-3-ols and proanthocyanidins from rose hip (*Rosa* sp.) species. J. Sep. Sci..

[68] Medveckienė B., Kulaitienė J., Levickienė D., Hallmann E. (2021). The effect of ripening stages on the accumulation of carotenoids, polyphenols and vitamin C in rosehip species/cultivars. Appl. Sci..

[69] Demir N., Yildiz O., Alpaslan M., Hayaloglu A.A. (2014). Evaluation of volatiles, phenolic compounds and antioxidant activities of rose hip (*Rosa* L.) fruits in Turkey. LWT.

[70] Nađpal J.D., Lesjak M.M., Šibul F.S., Anačkov G.T., Četojević-Simin D.D., Mimica-Dukić N.M., Beara I.N. (2016). Comparative study of biological activities and phytochemical composition of two rose hips and their preserves: *Rosa canina* L. and *Rosa arvensis* Huds. Food Chem..

[71] Yang Y., Lian G., Yu B. (2015). Naturally occurring polyphenolic glucosidase inhibitors. Israel J. Chem..

[72] Sales P.M., Souza P.M., Simeoni L.A., Magalhães P.O., Silveira D. (2012). α-Amylase inhibitors: A review of raw material and isolated compounds from plant source. J. Pharm. Pharmaceut. Sci..

[73] Gholamhoseinian A., Fallah H., Sharifi far F. (2009). Inhibitory effect of methanol extract of *Rosa damascena* Mill. flowers on α-glucosidase activity and postprandial hyperglycemia in normal and diabetic rats. Phytomedicine.

[74] Asghari B., Salehi P., Farimani M.M., Ebrahimi S.N. (2015). α-Glucosidase inhibitors from fruits of *Rosa canina* L.. Rec. Nat. Prod..

[75] Zhu J., Zhang B., Wang B., Li C., Fu X., Huang Q. (2019). In-vitro inhibitory effects of flavonoids in *Rosa roxburghii* and *R. sterilis* fruits on α-glucosidase: Effect of stomach digestion on flavonoids alone and in combination with acarbose. J. Funct. Foods.

[76] Jemaa H.B., Jemia A.B., Khlifi S., Ahmed H.B., Slama F.B., Benzarti A., Aouidet A. (2017). Antioxidant activity and α-amylase inhibitory potential of *Rosa canina* L.. Afr. J. Tradit. Compl. Altern. Med..

[77] Minekus M., Alminger M., Alvito P., Ballance S., Bohn T., Bourlieu C., Carrière F., Boutrou R., Corredig M., Dupont D. (2014). A standardised static in vitro digestion method suitable for food—An international consensus. Food Funct..

[78] Ochir S., Nishizawa M., Park B.J., Ishii K., Kanazawa T., Funaki M., Yamagishi T. (2010). Inhibitory effects of *Rosa gallica* on the digestive enzymes. J. Nat. Med..

[79] Farzaei F., Morovati M.R., Farjadmand F., Farzaei M.H. (2017). A mechanistic review on medicinal plants used for diabetes mellitus in Traditional Persian Medicine. J. Evid. Based Compl. Altern. Med..

[80] Bindu J., Narendhirakannan R.T. (2019). Role of medicinal plants in the management of diabetes mellitus: A review. 3 Biotech..

[81] Naveen Y.P., Urooj A., Byrappa K. (2021). A review on medicinal plants evaluated for anti-diabetic potential in clinical trials: Present status and future perspective. J. Herbal Med..

[82] Ochir S., Park B., Nishizawa M., Kanazawa T., Funaki M., Yamagishi T. (2010). Simultaneous determination of hydrolysable tannins in the petals of *Rosa rugosa* and allied plants. J. Nat. Med..

[83] Li H., Tanaka T., Zhang Y.-J., Yang C.-R., Kouno I. (2007). Rubusuaviins A–F, monomeric and oligomeric ellagitannins from chinese sweet tea and their α-amylase inhibitory activity. Chem. Pharm. Bull..

[84] Juśkiewicz J., Jurgonski A., Kołodziejczyk K., Kosmala M., Milala J., Zduńczyk Z., Fotschki B., Żary-Sikorska E. (2016). Blood glucose lowering efficacy of strawberry extracts rich in ellagitannins with different degree of polymerization in rats. Pol. J. Food Nutr. Sci..

[85] Adamczyk B., Salminen J.-P., Smolander A., Kitunen V. (2012). Precipitation of proteins by tannins: Effects of concentration, protein/tannin ratio and pH. Int. J. Food Sci. Technol..

[86] Hagerman A.E., Rice M.E., Ritchard N.T. (1998). Mechanisms of protein precipitation for two tannins, pentagalloyl glucose and epicatechin_16_(4→8) catechin (procyanidin). J. Agricult. Food Chem..

[87] Olennikov D.N. (2016). Ellagitannins and other phenolic compounds from *Comarum palustre*. Chem. Nat. Comp..

[88] Olennikov D.N., Kruglova M.Y. (2013). New quercetin glucoside and other phenolic compounds from *Filipendula* genus. Chem. Nat. Comp..

[89] Olennikov D.N., Kashchenko N.I. (2017). Spireasalicin, a new acylated glycoside of quercetin from *Spiraea salicicfolia*. Chem. Nat. Comp..

[90] Olennikov D.N., Chirikova N.K. (2020). New compounds from flowers of *Phlojodicarpus sibiricus*. Chem. Nat. Comp..

[91] Olennikov D.N., Kashchenko N.I. (2020). New *C*,*O*-glycosylflavones from the genus *Silene*. Chem. Nat. Comp..

[92] Olennikov D.N. (2020). New flavonoids from *Artemisia frigida*. Chem. Nat. Comp..

[93] Olennikov D.N., Chirikova N.K. (2021). New acylated flavone-*O*-glycosides and iridoids from the genus *Veronica*. Chem. Nat. Comp..

[94] Olennikov D.N., Chirikova N.K. (2021). New compounds from Siberian *Gentiana* species. II. Xanthone and *C*,*O*-glycosylflavone. Chem. Nat. Comp..

[95] Chirikova N.K., Olennikov D.N., Tankhaeva L.M. (2010). Quantitative determination of flavonoid content in the aerial part of Baical scullcap (*Scutellaria baicalensis* Georgi). Russ. J. Bioorg. Chem..

[96] Damien Dorman H.J., Shikov A.N., Pozharitskaya O.N., Hiltunen R. (2011). Antioxidant and pro-oxidant evaluation of a *Potentilla alba* L. rhizome extract. Chem. Biodiv..

[97] Sun B., Ricardo-da-Silva J.M., Spranger I. (1998). Critical factors of vanillin assay for catechins and proanthocyanidins. J. Agricult. Food Chem..

[98] Olennikov D.N., Tankhaeva L.M. (2010). Quantitative determination of phenolic compounds in *Mentha piperita* leaves. Chem. Nat. Comp..

[99] Porter L.J., Hrstich L.N., Chan B.G. (1986). The conversion of procyanidins and prodelphinidins to cyanidin and delphinidin. Phytochemistry.

[100] Wilson T.C., Hagerman A.E. (1990). Quantitative determination of ellagic acid. J. Agric. Food Chem..

[101] Inoue K.H., Hagerman A.E. (1988). Determination of gallotannin with rhodanine. Anal. Biochem..

[102] Olennikov D.N., Tankhaeva L.M., Samuelsen A.B. (2006). Quantitative analysis of polysaccharides from *Plantago major* leaves using the Dreywood method. Chem. Nat. Comp..

[103] Olennikov D.N., Kashchenko N.I., Chirikova N.K., Gornostai T.G., Selyutina I.Y., Zilfikarov I.N. (2017). Effect of low temperature cultivation on the phytochemical profile and bioactivity of Arctic plants: A case of *Dracocephalum palmatum*. Int. J. Mol. Sci..

[104] Rajković M.B., Novaković I.D., Petrović A. (2007). Determination of titratable acidity in white wine. J. Agricult. Sci..

[105] Iverson S.J., Lang S.L.C., Cooper M.H. (2001). Comparison of the Bligh and Dyer and Folch methods for total lipid determination in a broad range of marine tissue. Lipids.

[106] Olennikov D.N., Kashchenko N.I. (2014). Componential profile and amylase inhibiting activity of phenolic compounds from *Calendula officinalis* L. leaves. Sci. World J..

