# Supplementary material for: Metabolites of Prickly Rose: Chemodiversity and Digestive-Enzyme-Inhibiting Potential of Rosa acicularis and the Main Ellagitannin Rugosin D
Source: Plants (Basel). 2021 Nov 20;10(11):2525. doi: 10.3390/plants10112525 (PMC8622385; doi:10.3390/plants10112525)
Supplement: Supplementary file 1 [file plants-10-02525-s001.zip › plants-1471669-supplementary.pdf]

plants

Supplementary Material

Metabolites of Prickly Rose: Chemodiversity and Digestive-Enzyme-Inhibiting Potential of *Rosa acicularis* and the Main Ellagitannin Rugosin D

Daniil N. Olennikov [1,*], Vladimir V. Chemposov [2] and Nadezhda K. Chirikova [2]

[1] Laboratory of Medical and Biological Research, Institute of General and Experimental Biology, Siberian Division, Russian Academy of Science, 670047 Ulan-Ude, Russia
[2] Department of Biology, Institute of Natural Sciences, North-Eastern Federal University, 677027 Yakutsk, Russia; soulkage94@gmail.com (V.V.C.), hofnung@mail.ru (N.K.C.)
* Correspondence: olennikovdn@mail.ru; Tel.: +8-902-160-06-27

Content

Table S1. Reference standards and internal standards used for the qualitative and quantitative analysis by HPLC-MS and HPLC-DAD.

Table S2. Optimized MRM transitions of compounds **1–123** in HPLC-MS/MS analysis.

Table S3. Regression equations, correlation coefficients, standard deviation, limits of detection, limits of quantification and linear ranges for 34 reference standards.

Figure S1. HPLC-DAD chromatogramms of gastro-intestinal digestion markers after digestion of artificial substrate mixture.

Table S1. Reference standards and internal standards used for the qualitative and quantitative analysis by HPLC-MS and HPLC-DAD.

Compound (No in Table 2), Formula	Purity (≥), %	Manufacturer (cat. No) or Isolation Reference *	Used for Qualitative (QL) and Quantitative (QT) Analysis of Compounds (No in Table 2)
1-*O*-Galloyl-β-D-glucopyranose (**1**) 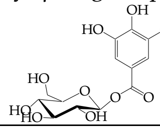	90	Sigma (69288)	QL: **1**. QT: **1**
Gallic acid (**2**) 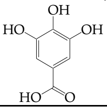	97	Sigma (G7384)	QL: **2**. QT: **2**
1-*O*-Caffeoylquinic acid (**3**) 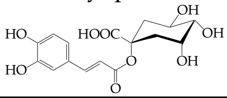	98	ChemFaces (CFN99121)	QL: **3**. QT: **3**, **7**
2-Pyrone-4,6-dicarboxylic acid (**8**) 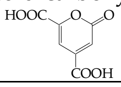	95	Lab collection/isolated from *Comarum palustre* [87]	QL: **8**. QT: **8**
Epigallocatechin (**9**) 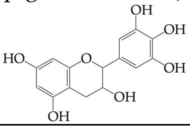	98	Extrasynthese (0979S)	QL: **9**. QT: **9**
5-*O*-Caffeoylquinic acid (**10**) 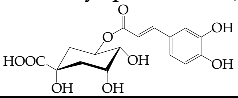	95	Sigma (C3878)	QL: **10**. QT: **10**
Catechin (**11**) 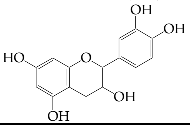	99	Extrasynthese (0976S)	QL: **11**. QT: **11**
1,6-Di-*O*-galloyl glucose (**13**) 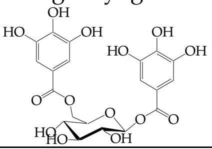	92	Toronto (D293195)	QL: **13**. QT: **12**, **13**
Tellimagrandin I_1 (**14**) 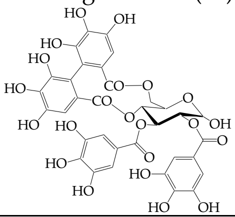	No data	Lab collection/isolated from *Filipendula ulmaria* [88]	QL: **14**
Epicatechin (**15**) 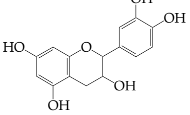	99	Extrasynthese (0977S)	QL: **15**. QT: **15**

Rugosin B_1 (**16**) 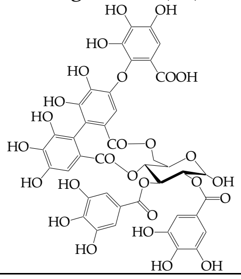	No data	Lab collection/isolated from *Filipendula ulmaria* [88]	QL: **16**
1,3,6-Tri-*O*-galloyl glucose (**18**) 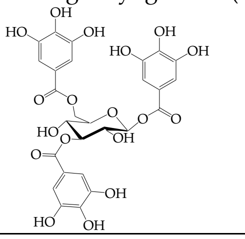	90	Sigma (78864)	QL: **18**. QT: **17**, **18**, **20**
Tellimagrandin I_2 (**19**) 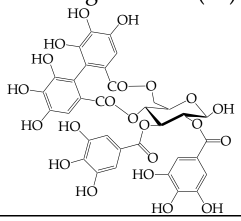	No data	Lab collection/isolated from *Filipendula ulmaria* [88]	QL: **19**
Tellimagrandin II (**21**) 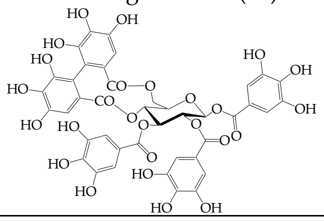	No data	Lab collection/isolated from *Filipendula ulmaria* [88]	QL: **21**
Rugosin B_2 (**22**) 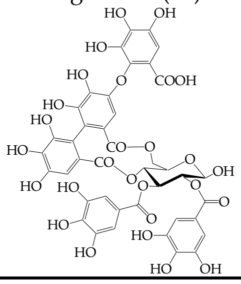	No data	Lab collection/isolated from *Filipendula ulmaria* [88]	QL: **22**

Rugosin E1 (**24**) 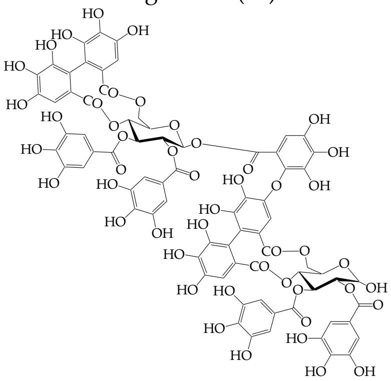	No data	Lab collection/isolated from *Filipendula ulmaria* [88]	QL: **24**
Rugosin E2 (**25**) 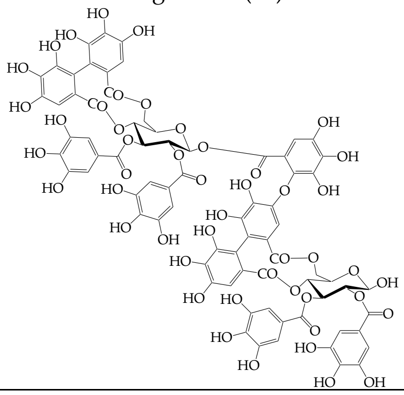	No data	Lab collection/isolated from *Filipendula ulmaria* [88]	QL: **25**
Rugosin A (**26**) 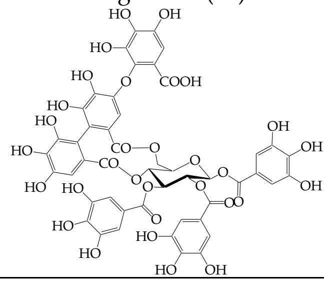	No data	Lab collection/isolated from *Filipendula ulmaria* [88]	QL: **26**
Rugosin D (**27**) 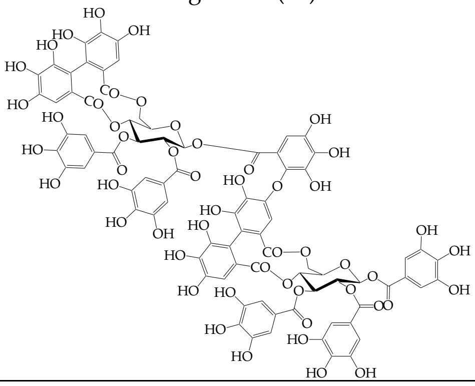	No data	Lab collection/isolated from *Filipendula ulmaria* [88]	QL: **27**
1-*O*-Ellagoyl-β-D-glucopyranoside (**28**) 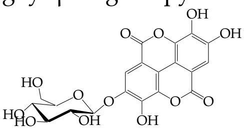	No data	Lab collection/isolated from *Punica granatum* [40]	QL: **28**

Ellagic acid (**29**) 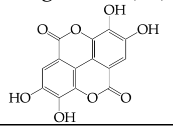	95	Sigma (E2250)	QL: **29**. QT: **6**, **29**, **30**, **31**, **36**, **39**, **43**, **44**, **90**, **91**, **94**, **110**, **112**, **113**
1,2,3,6-Tetra-*O*-galloyl glucose (**33**) 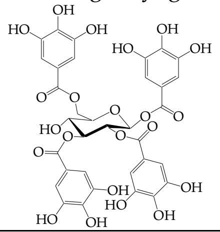	98	BioBioPha (BBPP05354)	QL: **33**. QT: **32**, **33**
1,2,3,4,6-Penta-*O*-galloyl glucose (**47**) 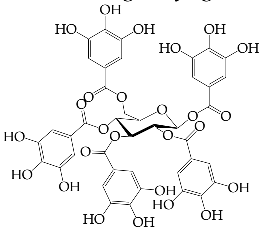	96	Sigma (67548)	QL: **47**. QT: **46**, **47**, **48**, **49**, **50**, **92**
Rutin = quercetin-3-*O*-rutinoside (**65**) 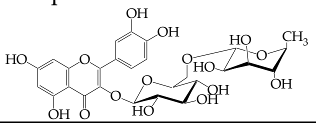	95	Sigma (R2303)	QL: **65**. QT: **52**, **54**, **57**, **61**, **66**, **67**, **65**
Miquelianin = quercetin-3-*O*-β-D-glucuronopyranoside (**68**) 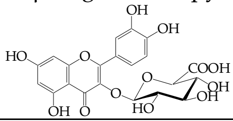	95	Sigma (00310590)	QL: **68**. QT: **68**
Kaempferol-3-*O*-β-D-glucuronopyranoside (**71**) 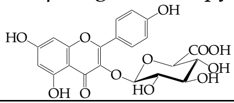	97	Sigma (79273)	QL: **71**. QT: **58**, **60**, **62**, 69, **70**, **71**
Quercitrin = quercetin-3-*O*-α-L-rhamnopyranoside (**72**) 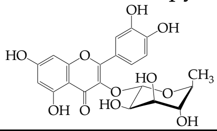	98	Sigma (740580)	QL: **72**. QT: **72**
Helichrysoside = quercetin-3-*O*-(6′′-*O*-*p*-coumaroyl)-β-D-glucopyranoside (**73**) 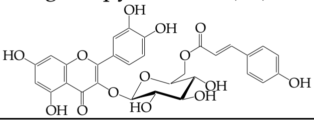	No data	Lab collection/isolated from *Spiraea salicicfolia* [89]	QL: **73**, **78**, **79**, **80**, **81**
Juglanin = kaempferol-3-*O*-α-L-arabinofuranoside (**74**) 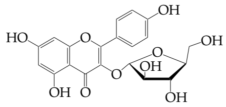	98	MCE (HY-N3442)	QL: **74**. QT: **74**

Tiliroside = kaempferol-3-*O*-(6′′-*O*-*p*-coumaroyl)-β-D-glucopyranoside (**75**) 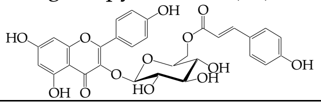	98	Sigma (79257)	QL: **75**. QT: **75**, **82**, **83**, **84**, **85**, **86**
Kaempferol-3-*O*-(6′′-galloyl)-β-D-glucopyranoside (**76**) 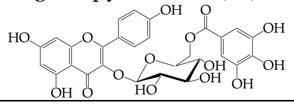	98	ChemFaces (CFN92383)	QL: **76**. QT: **59**, **76**, **77**
Peonidin 3,5-di-*O*-β-D-glucopyranoside (**93**) 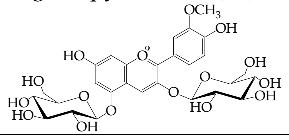	90	Extrasynthese (0918)	QL: **93**. QT: **93**
Procyanidin B_3 (**97**) 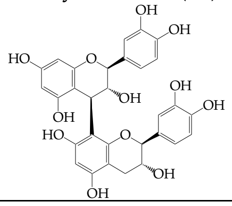	95	Carbosynth (FP65542)	QL: **97**. QT: **97**
Procyanidin B_1 (**98**) 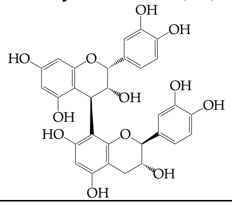	99	MCE (HY-N0795)	QL: **98**. QT: **34**, **35**, **37**, **38**, **40**, **41**, **42**, **45**, **95**, **96**, **98**, **99**, **101**, **102**, **103**, **104**, **114**, **115**, **116**, **117**, **118**, **119**, **120**, **121**, **122**, **123**
Procyanidin B_4 (**100**) 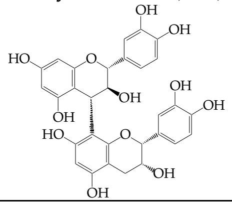	98	ChemFaces (CFN91171)	QL: **100**. QT: **100**
Procyanidin B_2 (**105**) 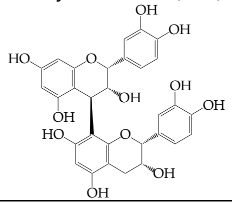	90	Sigma (42157)	QL: **105**. QT: **105**
Procyanidin B_2 3-*O*-gallate (**106**) 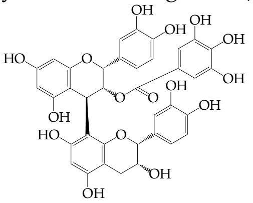	95	TransMIT (C6201-P044)	QL: **106**. QT: **106**, **108**

Procyanidin B_2 3′′-*O*-gallate (**107**) 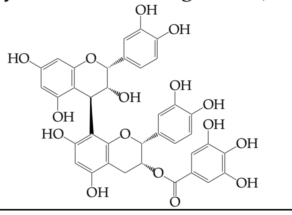	97	ALB (ALB-RS-9598)	QL: **107**. QT: **107**
Procyanidin B_2 3,3′′-di-*O*-gallate (**109**) 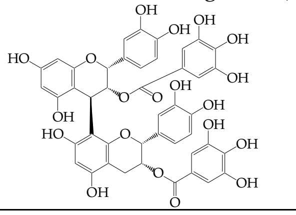	97	BOC (79907-44-1)	QL: **109**. QT: **109**
Amritoside = 1-*O*-ellagoyl-gentiobiose (**110**) 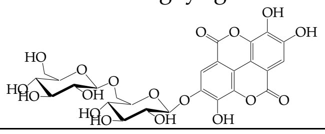	No data	Lab collection/isolated from *Punica granatum* [40]	QL: **110**
Epicatechin gallate (**111**) 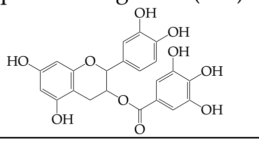	97.5	Extrasynthese (0978S)	QL: **111**. QT: **111**
Eschweilenol C = ellagic acid 4-*O*-α-L-rhamnopyranoside (**113**) 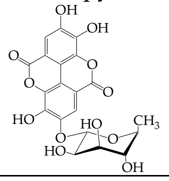	98	ChemFaces (CFN95363)	QL: **113**. QT: **113**
Corilagin 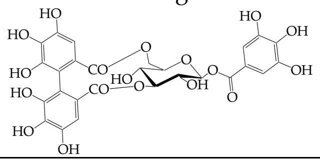	98	Sigma (G0424)	QT: **4**, **5**, **14**, **16**, **19**, **21**, **22**, **23**, **24**, **25**, **26**, **27**, **87**, **88**, **89**
Taxifolin = dihydroquercetin 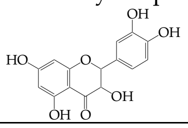	95	Sigma (PHL89284)	QT: **51**, **53**, **55**, **63**, **64**
Quercetin-3-*O*-(6′′-galloyl)-β-D-galactopyranoside 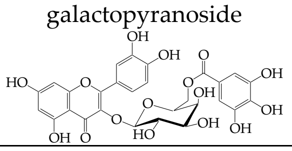	98	MCE (HY-N7024)	QT: **56**
3′,4′-Di-*O*-acetyl-*cis*-khellactone 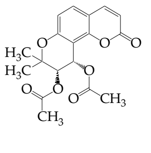	No data	Lab collection/isolated from *Phlojodicarpus sibiricus* [90]	Internal standard 1

Isovitexin-7,2″-di-*O*-glucoside 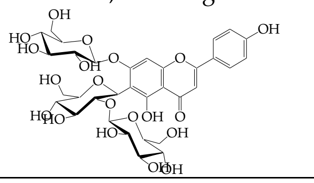	No data	Lab collection/isolated from *Silene dioica* [91]	Internal standard 2
Isojaceoside 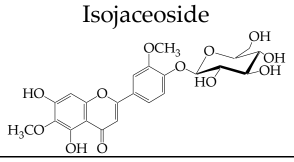	No data	Lab collection/isolated from *Artemisia frigida* [92]	Internal standard 3
6-*O*-Sinapoyl catalpol 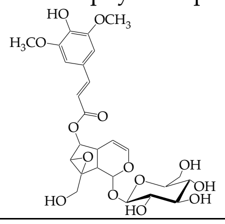	No data	Lab collection/isolated from *Veronica longifolia* [93]	Internal standard 4
Neomangiferin 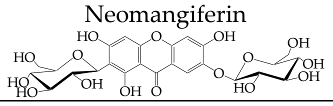	No data	Lab collection/isolated from *Gentiana daurica* [94]	Internal standard 5
p-Nitrophenol 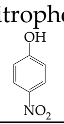	99	Sigma (241326)	Marker of ethylidene-*p*-nitrophenyl-α-D-maltoheptaoside digestion
7-Amino-4-methylcoumarin 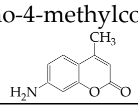	99	Sigma (257370)	Marker of N_α-benzoyl-L-arginine-7-amino-4-methylcoumarin hydrochloride digestion
4-Methylumbelliferone 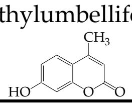	98	Sigma (M1381)	Marker of 4-methylumbelliferyl heptanoate digestion

* Manufacturers list: ALB—ALB Materials Inc (Henderson, NV, USA); BioBioPha—BioBioPha (Kunming, Yunnan, PRC); BOC—BOC Sciences (Shirley, NY, USA); Carbosynth—Carbosynth Ltd (Compton, Great Britain); ChemFaces—ChemFaces (Wuhan, Hubei, PRC); Extrasynthese—Extrasynthese (Lyon, France); MCE—MCE Med Chem Express (Monmouth, NJ, USA); Sigma—Sigma-Aldrich (St. Louis, MO, USA); Toronto—Toronto Research Chemicals (North York, ON, Canada); TransMIT—TransMIT GmbH (Gießen, Germany).

Table S2. Optimized MRM transitions of compounds **1–123** in HPLC-MS/MS analysis.

No	Compound	Ionization	Precur-sor	Quan-tifier
**1**	1-*O*-Galloyl glucose (glucogallin)	Negative	331	169
**2**	Gallic acid	Negative	169	125
**3**	1-*O*-Caffeoylquinic acid	Negative	353	135
**4**	Galloyl-hexahydroxydiphenoyl-di-*O*-hexoside	Negative	795	301
**5**	Galloyl-hexahydroxydiphenoyl-di-*O*-hexoside	Negative	795	301
**6**	Ellagic acid tri-*O*-hexoside	Negative	787	301
**7**	Caffeic acid *O*-hexoside	Negative	341	179
**8**	2-Pyrone-4,6-dicarboxylic acid	Negative	183	111
**9**	Epigallocatechin	Negative	305	137
**10**	5-*O*-Caffeoylquinic acid	Negative	353	165
**11**	Catechin	Negative	289	137
**12**	Di-*O*-galloyl hexose	Negative	483	169
**13**	1,6-Di-*O*-galloyl glucose	Negative	483	125
**14**	Tellimagrandin I_1	Negative	392	301
**15**	Epicatechin	Negative	289	137
**16**	Rugosin B_1	Negative	476	301
**17**	Tri-*O*-galloyl hexose	Negative	635	169
**18**	1,3,6-Tri-*O*-galloyl glucose	Negative	635	169
**19**	Tellimagrandin I_2	Negative	392	301
**20**	Tri-*O*-galloyl hexose	Negative	635	169
**21**	Tellimagrandin II_1	Negative	468	301
**22**	Rugosin B_2	Negative	476	301
**23**	Tellimagrandin II_2	Negative	468	301
**24**	Rugosin E_1	Negative	860	301
**25**	Rugosin E_2	Negative	860	301
**26**	Rugosin A	Negative	552	301
**27**	Rugosin D	Negative	623	301
**28**	1-*O*-Ellagoyl glucose	Negative	463	301
**29**	Ellagic acid	Negative	301	185
**30**	Ellagic acid methyl ester *O*-hexoside	Negative	477	301
**31**	Ellagic acid methyl ester *O*-hexoside	Negative	477	301
**32**	Tetra-*O*-galloyl hexose	Negative	787	169
**33**	1,2,3,6-Tetra-*O*-galloyl glucose	Negative	787	169
**34**	Epicatechin / catechin trimer	Negative	865	451
**35**	Epicatechin / catechin trimer	Negative	865	451
**36**	Ellagic acid dimethyl ester *O*-hexoside	Negative	491	329
**37**	Epicatechin / catechin trimer	Negative	865	577
**38**	Epicatechin / catechin trimer	Negative	865	577
**39**	Di-ellagoyl methyl ester *O*-hexoside	Negative	761	477
**40**	Epicatechin / catechin tetramer	Negative	1153	577
**41**	Epicatechin / catechin tetramer	Negative	1153	577

**42**	Epicatechin / catechin tetramer	Negative	1153	577
**43**	Di-ellagoyl dimethyl ester *O*-hexoside	Negative	775	477
**44**	Ellagic acid trimethyl ester *O*-hexoside	Negative	505	343
**45**	Epicatechin / catechin tetramer	Negative	1153	863
**46**	Penta-*O*-galloyl hexose	Negative	939	787
**47**	1,2,3,4,6-Penta-*O*-galloyl glucose	Negative	939	787
**48**	Hexa-*O*-galloyl hexose	Negative	1091	787
**49**	Hexa-*O*-galloyl hexose	Negative	1091	787
**50**	Hexa-*O*-galloyl hexose	Negative	1091	787
**51**	Dihydroquercetin di-*O*-hexuronoside-di-*O*-hexoside	Negative	979	817
**52**	Quercetin di-*O*-hexuronoside-tri-*O*-hexoside	Negative	1139	815
**53**	Dihydroquercetin *O*-hexuronoside-di-*O*-hexoside	Negative	803	641
**54**	Quercetin *O*-hexuronoside-tri-*O*-hexoside	Negative	963	801
**55**	Dihydroquercetin *O*-hexuronoside-*O*-hexoside	Negative	641	479
**56**	Quercetin *O*-hexuronoside-tri-*O*-hexoside-*O*-gallate	Negative	1115	639
**57**	Quercetin di-*O*-hexuronoside-di-*O*-hexoside	Negative	977	653
**58**	Kaempferol *O*-hexuronoside-tri-*O*-hexoside	Negative	947	623
**59**	Kaempferol *O*-hexuronoside-tri-*O*-hexoside-*O*-gallate	Negative	1099	623
**60**	Kaempferol di-*O*-hexuronoside-di-*O*-hexoside	Negative	961	461
**61**	Quercetin *O*-hexuronoside-di-*O*-hexoside	Negative	801	477
**62**	Kaempferol *O*-hexuronoside-di-*O*-hexoside	Negative	785	461
**63**	Dihydroquercetin *O*-hexoside	Negative	465	303
**64**	Dihydroquercetin *O*-hexuronoside	Negative	479	303
**65**	Quercetin-*O*-rutinoside (rutin)	Negative	609	463
**66**	Quercetin *O*-hexuronoside-*O*-hexoside	Negative	639	477
**67**	Quercetin *O*-hexuronoside-*O*-pentoside	Negative	609	477
**68**	Quercetin-3-*O*-glucuronide (miquelianin)	Negative	477	301
**69**	Kaempferol *O*-hexuronoside-*O*-hexoside	Negative	623	461
**70**	Kaempferol *O*-hexuronoside-*O*-pentoside	Negative	593	461
**71**	Kaempferol-3-*O*-glucuronide	Negative	461	285
**72**	Quercetin-3-*O*-rhamnoside (quercitrin)	Negative	447	301
**73**	Quercetin-3-*O*-(6′′-*O*-*p*-coumaroyl)-glucoside (helichrysoside)	Negative	609	463
**74**	Kaempferol-3-*O*-arabinoside (juglanin)	Negative	417	285
**75**	Kaempferol-3-*O*-(6′′-*O*-*p*-coumaroyl)-glucoside (tiliroside)	Negative	593	447
**76**	Kaempferol-3-*O*-(6′′-*O*-galloyl)-glucoside	Negative	599	447
**77**	Kaempferol *O*-hexuronoside-*O*-gallate	Negative	613	461
**78**	Quercetin *O*-hexoside-*O*-*p*-coumarate-*O*-gallate	Negative	761	609
**79**	Quercetin *O*-hexuronoside-*O*-*p*-coumarate-*O*-gallate	Negative	775	623
**80**	Quercetin *O*-hexoside-di-*O*-*p*-coumarate	Negative	755	463
**81**	Quercetin *O*-hexuronoside-di-*O*-*p*-coumarate	Negative	769	477
**82**	Kaempferol *O*-hexuronoside-*O*-*p*-coumarate-*O*-gallate	Negative	759	607
**83**	Kaempferol *O*-hexuronoside-*O*-hexoside-di-*O*-*p*-coumarate	Negative	915	461
**84**	Kaempferol *O*-hexoside-di-*O*-*p*-coumarate	Negative	739	447

**85**	Kaempferol *O*-hexuronoside-di-*O*-*p*-coumarate	Negative	753	461
**86**	Kaempferol *O*-hexuronoside-tri-*O*-*p*-coumarate	Negative	899	461
**87**	Hexahydroxydiphenoyl-tri-*O*-galloyl-hexose (tellimagrandin II isomer)	Negative	468	301
**88**	Valoneoyl-tri-*O*-galloyl-hexose (rugosin A isomer)	Negative	552	301
**89**	Hexahydroxydiphenoyl-valoneoyl-tetra-*O*-galloyl-di-*O*-hexose (rugosin D isomer)	Negative	623	765
**90**	Ellagic acid *O*-hexoside	Negative	463	301
**91**	Epicatechin / catechin tetramer	Negative	1153	577
**92**	Hexa-*O*-galloyl hexoside	Negative	1091	635
**93**	Peonidin 3,5-di-*O*-glucoside	Positive	625	301
**94**	Ellagic acid tri-*O*-hexoside	Negative	787	463
**95**	Epicatechin / catechin dimer *O*-hexoside	Negative	739	451
**96**	Epicatechin / catechin dimer *O*-hexoside	Negative	739	451
**97**	Procyanidin B_3	Negative	577	407
**98**	Procyanidin B_1	Negative	577	407
**99**	Epicatechin / catechin dimer *O*-hexoside	Negative	739	451
**100**	Procyanidin B_4	Negative	577	407
**101**	Epicatechin / catechin dimer *O*-hexoside	Negative	739	451
**102**	Epicatechin / catechin dimer *O*-hexoside	Negative	739	451
**103**	Epicatechin / catechin dimer *O*-hexoside	Negative	739	451
**104**	Epicatechin / catechin dimer *O*-hexoside	Negative	739	451
**105**	Procyanidin B_2	Negative	577	407
**106**	Procyanidin B_2 3-*O*-gallate	Negative	729	441
**107**	Procyanidin B_2 3′′-*O*-gallate	Negative	729	441
**108**	Epicatechin / catechin dimer *O*-gallate	Negative	729	441
**109**	Procyanidin B_2 3,3′′-di-*O*-gallate	Negative	881	559
**110**	1-*O*-Ellagoyl-gentiobiose (amritoside)	Negative	625	301
**111**	Epicatechin gallate	Negative	441	289
**112**	Ellagic acid di-*O*-desoxyhexoside	Negative	593	301
**113**	Ellagic acid 4-*O*-rhamnoside (eschweilenol C)	Negative	447	301
**114**	Epicatechin / catechin tetramer	Negative	1153	577
**115**	Epicatechin / catechin tetramer	Negative	1153	577
**116**	Epicatechin / catechin tetramer	Negative	1153	577
**117**	Epicatechin / catechin tetramer	Negative	1153	577
**118**	Epicatechin / catechin pentamer	Negative	1441	863
**119**	Epicatechin / catechin pentamer	Negative	1441	863
**120**	Epicatechin / catechin pentamer	Negative	1441	863
**121**	Epicatechin / catechin pentamer	Negative	1441	863
**122**	Epicatechin / catechin pentamer	Negative	1441	863
**123**	Epicatechin / catechin pentamer	Negative	1441	863

Table S3. Regression equations, correlation coefficients (r^2), standard deviation (S_{YX}), limits of detection (LOD), limits of quantification (LOQ) and linear ranges for 34 reference standards.

Compound	Ionization [a]	CE [b] (eV)	Regression Equation [c]		r^2	S_{YX}	LOD/ LOQ (μg/mL)	Linear Range (μg/mL)
			a	$b \cdot 10^6$				
1-*O*-Caffeoylquinic acid	N	-15	2.5394	-1.2360	0.9994	$0.45 \cdot 10^{-2}$	0.006/0.02	0.02–300.0
1-*O*-Galloyl-β-D-glucopyranose	N	-20	1.3586	-0.0663	0.9987	$9.69 \cdot 10^{-2}$	0.24/0.71	0.8–100.0
1,6-Di-*O*-galloyl glucose	N	-20	1.7552	-0.0569	0.9982	$8.89 \cdot 10^{-2}$	0.18/0.51	0.6–100.0
1,3,6-Tri-*O*-galloyl glucose	N	-25	2.1064	-0.0499	0.9985	$8.74 \cdot 10^{-2}$	0.14/0.42	0.5–100.0
1,2,3,6-Tetra-*O*-galloyl glucose	N	-25	2.0168	-0.0629	0.9973	$9.00 \cdot 10^{-2}$	0.15/0.45	0.5–100.0
1,2,3,4,6-Penta-*O*-galloyl glucose	N	-25	2.4561	-0.0171	0.9979	$12.33 \cdot 10^{-2}$	0.17/0.50	0.6–100.0
2-Pyrone-4,6-dicarboxylic acid	N	-20	1.8535	0.0742	0.9970	$4.55 \cdot 10^{-2}$	0.08/0.25	0.3–100.0
5-*O*-Caffeoylquinic acid	N	-15	2.9021	-1.4184	0.9998	$0.39 \cdot 10^{-2}$	0.004/0.01	0.02–300.0
Catechin	N	-20	0.9562	-0.0521	0.9971	$7.79 \cdot 10^{-2}$	0.27/0.82	0.90–100.0
Corilagin	N	-35	0.9634	-0.3518	0.9804	$7.34 \cdot 10^{-2}$	0.25/0.76	0.8–100.0
Gallic acid	N	-10	2.6538	-0.1376	0.9990	$1.17 \cdot 10^{-2}$	0.01/0.04	0.10–100.0
Epicatechin	N	-20	1.0828	-0.0456	0.9973	$6.85 \cdot 10^{-2}$	0.21/0.63	0.70–100.0
Epicatechin gallate	N	-20	1.5152	-0.0523	0.9979	$12.67 \cdot 10^{-2}$	0.28/0.84	0.90–100.0
Epigallocatechin	N	-35	1.2824	-0.1440	0.9987	$14.44 \cdot 10^{-2}$	0.37/1.12	1.2–100.0
Ellagic acid	N	-30	0.9114	-0.6312	0.9887	$6.37 \cdot 10^{-2}$	0.23/0.70	0.7–100.0
Eschweilenol C	N	-30	1.2820	-0.9634	0.9697	$11.64 \cdot 10^{-2}$	0.30/0.91	1.0–100.0
Helichrysoside	N	-30	2.0319	-0.3615	0.9811	$10.09 \cdot 10^{-2}$	0.17/0.52	0.6–100.0
Juglanin	N	-20	2.0384	-0.3640	0.9975	$2.02 \cdot 10^{-2}$	0.03/0.10	0.10–350.0
Kaempferol-3-*O*-β-D-glucurono pyranoside	N	-30	2.2126	-0.5160	0.9987	$8.11 \cdot 10^{-2}$	0.12/0.37	0.4–100.0
Kaempferol-3-*O*-(6′′-galloyl)-β-D-glucopyranoside	N	-10	1.2416	-0.3615	0.9901	$3.02 \cdot 10^{-2}$	0.08/0.24	0.3–100.0
Miquelianin	N	-20	1.6705	-0.4374	0.9988	$12.79 \cdot 10^{-2}$	0.25/0.77	0.8–100.0
Peonidin 3,5-di-*O*-β-D-glucopyranoside	P	+20	0.9634	-0.8634	0.9832	$10.37 \cdot 10^{-2}$	0.36/1.07	1.1–100.0
Procyanidin B_1	N	-30	1.3722	-0.0829	0.9973	$9.93 \cdot 10^{-2}$	0.24/0.72	0.80–100.0
Procyanidin B_2	N	-30	1.3620	-0.0820	0.9961	$9.91 \cdot 10^{-2}$	0.21/0.72	0.80–100.0
Procyanidin B_2 3-*O*-gallate	N	-35	0.9361	-0.4518	0.9870	$9.35 \cdot 10^{-2}$	0.32/1.00	1.0–100.0
Procyanidin B_2 3′′-*O*-gallate	N	-35	1.5963	-0.3518	0.9910	$14.63 \cdot 10^{-2}$	0.30/0.92	1.0–100.0
Procyanidin B_2 3,3′′-di-*O*-gallate	N	-25	1.4632	-0.0524	0.9953	$8.12 \cdot 10^{-2}$	0.18/0.56	0.6–100.0
Procyanidin B_3	N	-20	1.6341	-0.4283	0.9900	$15.02 \cdot 10^{-2}$	0.30/0.92	1.0–100.0
Procyanidin B_4	N	-25	1.0634	-0.0933	0.9902	$10.01 \cdot 10^{-2}$	0.31/0.94	1.0–100.0
Quercitrin	N	-20	1.9871	-0.6871	0.9984	$5.63 \cdot 10^{-2}$	0.09/0.28	0.40–400.0
Quercetin-3-*O*-(6′′-galloyl)-β-D-galactopyranoside	N	-25	1.1492	-0.6010	0.9980	$4.68 \cdot 10^{-2}$	0.14/0.41	0.50–400.0
Rutin	N	-20	1.2716	-0.7389	0.9897	$9.14 \cdot 10^{-2}$	0.23/0.72	0.80–400.0
Tiliroside	N	-30	2.3312	-0.4563	0.9803	$14.92 \cdot 10^{-2}$	0.21/0.64	0.7–100.0
Taxifolin	N	-15	1.1105	-0.3211	0.9937	$4.18 \cdot 10^{-2}$	0.12/0.38	0.4–100.0

[a] Ionization mode : N—negative ; P—positive. [b] CE—collision energy. [c] Regression equation: $y = a \cdot x + b$.

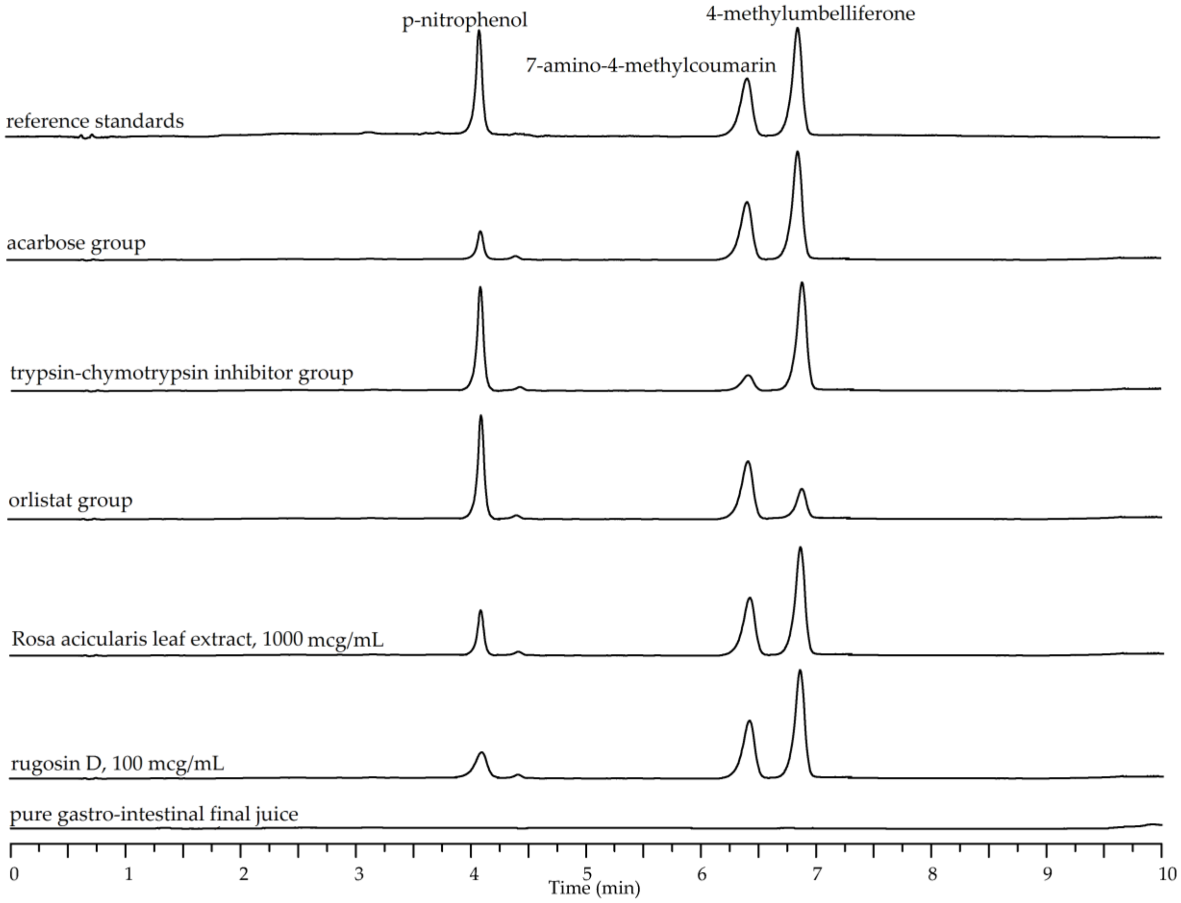

Figure S1. HPLC-DAD chromatogramms of gastro-intestinal digestion markers (*p*-nitrophenol, 7-amino-4-methylcoumarin, 4-methylumbelliferone) after digestion of artificial substrate mixture. Experimental groups: reference standards (*p*-nitrophenol, 20 µg/mL; 7-amino-4-methylcoumarin, 10 µg/mL; 4-methylumbelliferone, 15 µg/mL); acarbose group; trypsin-chymotrypsin inhibitor group; orlistat group; *R. acivularis* leaf extract, 1000 µg/mL, group; rugosin D, 100 µg/mL, group; pure gastro-intestinal final juice without addition of plant extracts and inhibitors.
